# Supramolecular Nano‐Tracker for Real‐Time Tracking of Drug Release and Efficient Combination Therapy

**DOI:** 10.1002/advs.202404731

**Published:** 2024-07-28

**Authors:** Xi Chen, Fang‐Yuan Chen, Yi Lu, Qiushi Li, Shujie Li, Chunxiong Zheng, Yadan Zheng, Lin Dang, Ru‐Yi Li, Yang Liu, Dong‐Sheng Guo, Shao‐Kai Sun, Zhanzhan Zhang

**Affiliations:** ^1^ School of Medical Imaging Tianjin Key Laboratory of Functional Imaging Tianjin Medical University Tianjin 300203 China; ^2^ College of Chemistry Key Laboratory of Functional Polymer Materials (Ministry of Education) State Key Laboratory of Medicinal Chemical Biology Nankai University Tianjin 300071 China; ^3^ School of Chemistry South China Normal University Guangzhou 510006 China; ^4^ College of Veterinary Medicine Northeast Agricultural University Harbin 150030 PR China; ^5^ Precision Medicine Center Tianjin Medical University General Hospital Tianjin 300000 China

**Keywords:** combination therapy, host‐guest interaction, hypoxic, non‐covalent, real‐time tracking

## Abstract

Real‐time tracking of drug release from nanomedicine in vivo is crucial for optimizing its therapeutic efficacy in clinical settings, particularly in dosage control and determining the optimal therapeutic window. However, most current real‐time tracking systems require a tedious synthesis and purification process. Herein, a supramolecular nano‐tracker (SNT) capable of real‐time tracking of drug release in vivo based on non‐covalent host‐guest interactions is presented. By integrating multiple cavities into a single nanoparticle, SNT achieves co‐loading of drugs and probes while efficiently quenching the photophysical properties of the probe through host‐guest complexation. Moreover, SNT is readily degraded under hypoxic tumor tissues, leading to the simultaneous release of drugs and probes and the fluorescence recovery of probes. With this spatial and temporal consistency in drug loading and fluorescence quenching, as well as drug release and fluorescence recovery, SNT successfully achieves real‐time tracking of drug release in vivo (Pearson r = 0.9166, *R* ^2^ = 0.8247). Furthermore, the released drugs can synergize effectively with fluorescent probes upon light irradiation, achieving potent chemo‐photodynamic combination therapy in 4T1‐bearing mice with a significantly improved survival rate (33%), providing a potential platform to significantly advance the development of nanomedicine and achieve optimal therapeutic effects in the clinic.

## Graphical abstract

Supramolecular nano‐tracker (SNT) is developed for real‐time tracking of drug release in vivo. SNT achieves non‐covalent drugs/probes loading and quenches probe fluorescence under physiological conditions, while releasing cargoes and restoring the probe fluorescence in tumor. Moreover, SNT facilitates chemo‐photodynamic‐based combination therapy upon light irradiation, demonstrating its potential for real‐time tracking of drug release and combination therapy.

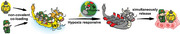

## 1 Introduction

Nanoparticle‐based drug delivery systems (nanoDDS) have undergone decades of development and are extensively utilized in clinical applications to improve therapeutic efficacy while mitigating the toxic side effects of drugs.^[^ ^1^ ^]^ Despite the notable success of nanoDDS, specific fundamental issues still need to be solved, such as the inability to track actual drug concentrations released from nanoDDS in targeted tissue.^[^ ^2^ ^]^ This knowledge gap poses significant challenges for physicians regarding dosage control and determining the optimal therapeutic window.^[^ ^3^ ^]^ Therefore, achieving real‐time tracking of drug release from nanoDDS in vivo is a critical requirement for improving drug bioavailability and optimizing the therapeutic efficacy of nanoDDS in clinical settings.^[^ ^4^ ^]^ Optical imaging, utilizing fluorescence molecules as probes, emerges as a promising option for tracking drug release due to its extremely high sensitivity and resolution.^[^ ^5^ ^]^ Currently, most fluorescence‐based drug tracking systems rely on developing photocaged compounds^[^ ^6^ ^]^ or utilizing fluorescence resonance energy transfer (FRET)^[^ ^7^ ^]^ effect between the acceptor (drugs) and the donor (fluorescence probe‐integrated nanoDDS) for monitoring. Despite their effectiveness, these strategies typically require complex personalized molecular designs for individual drugs.^[^ ^8^ ^]^ The time‐consuming synthesis and purification processes have greatly impeded their widespread application.^[^ ^6^, ^9^ ^]^ Therefore, an innovative real‐time tracking strategy generally applicable to various drugs without complicated synthesis processes is essential for nanoDDS to achieve optimal therapeutic efficacy in clinical settings.

In recent decades, supramolecular receptors, including calixarene,^[^ ^10^ ^]^ cyclodextrin,^[^ ^11^ ^]^ cucurbituril^[^ ^12^ ^]^ and pillararene,^[^ ^13^ ^]^ have gained significant attention in biomedical applications.^[^ ^14^ ^]^ Their characteristic cavitands and well‐defined molecular structure enable these receptors to accommodate a variety of guest molecules (drugs or probes) dynamically and reversibly through non‐covalent host‐guest interactions.^[^ ^15^ ^]^ Notably, the fluorescence and photoactivity of the probes are effectively quenched upon binding with some receptors, e.g., calixarenes, through a photoinduced electron transfer mechanism, while their photophysical properties are faithfully restored once released from the receptors.^[^ ^16^ ^]^ These unique characteristics of supramolecular receptors provide a facile, non‐covalent strategy for real‐time tracking of the drug release process in vivo.^[^ ^17^ ^]^ However, the defined binding stoichiometry (mostly 1:1) between supramolecular receptors and guest molecules makes it challenging to simultaneously load drugs and fluorescence probes into a single receptor, thus restricting their application in real‐time tracking.^[^ ^18^ ^]^ Therefore, a feasible approach for co‐loading fluorescence probes and drugs is crucial for utilizing supramolecular receptor‐based vectors in real‐time tracking of drug release.

**Figure 1:**
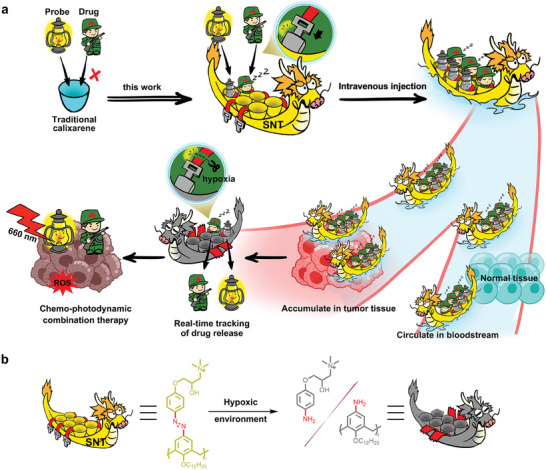
Schematic representation of SNT for real‐time tracking of drug release and effective chemo‐photodynamic combination therapy. a) Different from conventional supramolecular receptors that possess only one cavity, SNT integrates multiple cavities into a single nanoparticle and achieves co‐loading of drugs and probes. Upon reaching hypoxic TME, SNT gets effectively reduced by bio‐reductive enzymes, resulting in real‐time tracking of drug release and potent chemo‐photodynamic combination upon light irradiation. b) Corresponding hypoxia‐responsive mechanism.

Here, we present a supramolecular nano‐tracker (SNT) designed explicitly for the co‐loading of drugs and fluorescence probes, facilitating real‐time tracking of drug release. As illustrated in **Figure** **1**, SNT is facilely prepared through the co‐assembly of amphiphilic polymers, 4‐(dodecyloxy)benzamido‐terminated methoxy poly(ethylene glycol) (PEG‐12C), and supramolecular receptor quaternary‐ammonium‐modified azocalix[4]arene dodecyloxy ether (QAAC4A‐12C). This co‐assembling structure ingeniously integrates multiple cavities into a single ensemble, allowing the SNT to load drugs and probes simultaneously into a unified ensemble through host‐guest interactions. Notably, QAAC4A‐12C a hypoxia‐responsive supramolecular receptor, demonstrating high binding affinity toward various drugs and fluorescence probes. This property facilitates the co‐loading of drugs and fluorescence probes while efficiently quenching the photophysical properties of probes through complexation under physiological conditions.^[^ ^16^, ^17^ ^]^ Upon reaching hypoxic tumor microenvironment (TME), QAAC4A‐12C in SNT is readily reduced by reductase into aminocalix[4]arene pentadodecly ether (NH_2_C4A‐12C), which has low affinity for drugs and probes. This results in the spontaneous release of loaded cargoes and the fluorescence recovery of probes. With this spatial and temporal consistency in drug loading and fluorescence quenching, as well as drug release and fluorescence recovery, SNT successfully achieves real‐time tracking of drug release in vivo. Moreover, the released drugs synergize effectively with fluorescent probes upon light irradiation, achieving a potent chemo‐photodynamic combination therapy for tumors. This work utilized sulfonated zinc phthalocyanine (ZnPcS_4_) fluorescent probes, while NLG919 and paclitaxel (PTX) were employed as model drugs. The SNT effectively co‐delivered fluorescent probes and drugs to tumors, demonstrating real‐time tracking of drug release and successful chemo‐photodynamic combination therapy in 4T1 tumor‐bearing mice. These results highlight the potential of SNT as a platform for comprehending actual drug concentrations released from nanoDDS, with implications for achieving optimal therapeutic effects in clinical settings.

## 2 Results and Discussion

### 2.1 Structural Characterization of SNT

An ideal supramolecular system for real‐time tracking of drug release should fulfill the following criteria: i) being generally applicable to various drugs and fluorescence probes; ii) maintaining high structural integrity during blood circulation to prevent premature payload leakage and undesired fluorescence recovery of probes; iii) achieving synchronous release of drugs and fluorescence probes upon entering tumor tissues. Considering that hypoxia is a typical indicator of most solid tumors caused by abnormal proliferative and metabolic cell behaviors,^[^ ^19^ ^]^ a hypoxia‐responsive supramolecular receptor, QAAC4A‐12C, was rationally designed as the building block of SNT to facilitate precise loading of both drugs and fluorescence probes. In the synthesis of QAAC4A‐12C, NH_2_C4A‐12C was used as the supramolecular scaffold due to its amphiphilic nature and ease of modification (Figure S1, Supporting Information).^[^ ^20^ ^]^ Subsequently, hypoxia‐responsive azophenyl and positive‐charged quaternary ammonium groups were introduced into the upper rim of NH_2_C4A‐12C to create QAAC4A‐12C with improved affinity for a variety of hydrophobic and negative‐charged guests (Figure S2, Supporting Information).^[^ ^21^ ^]^

**Figure 2:**
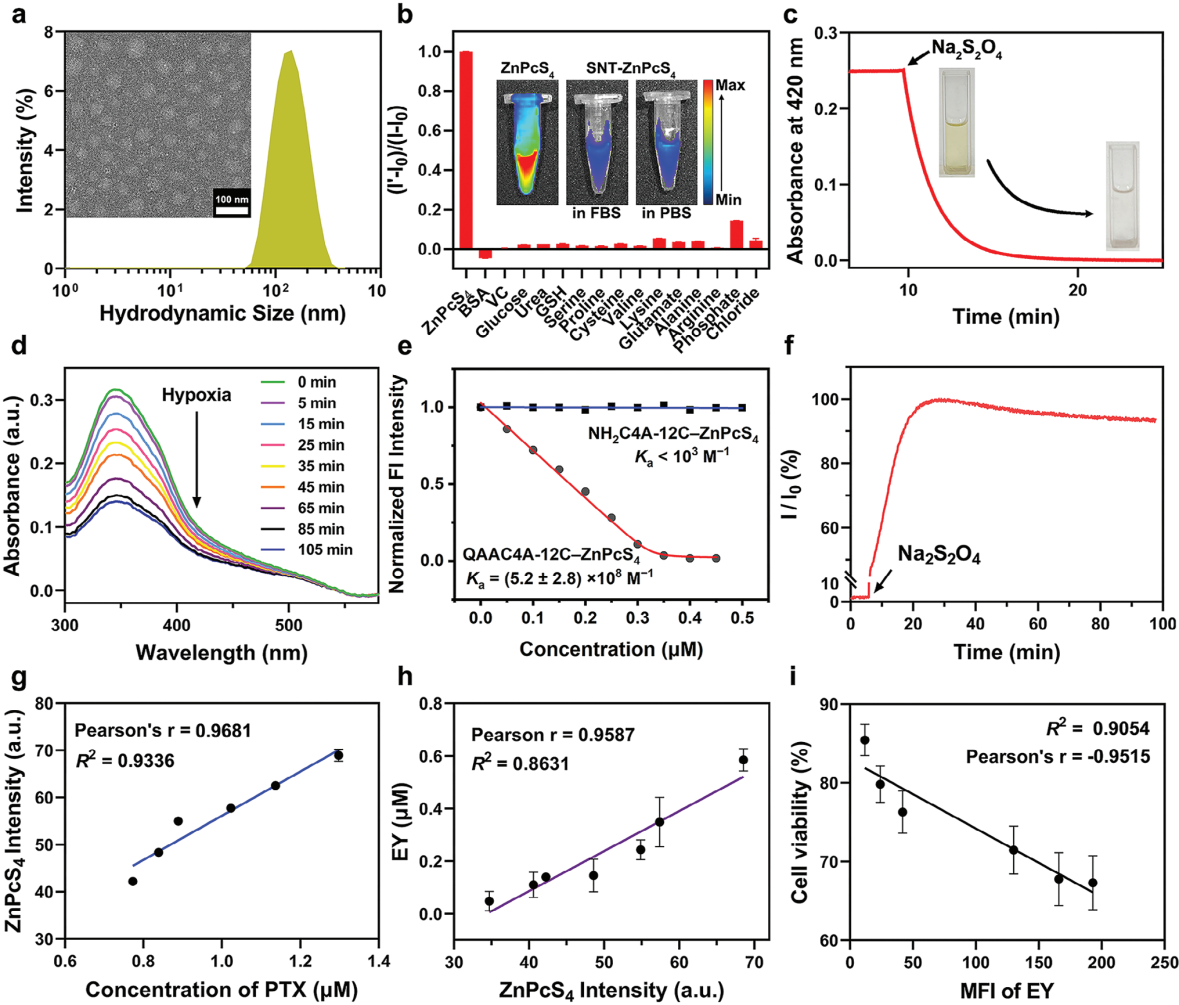
Characterization of SNT. a) The hydrodynamic size and TEM image (inset) of SNT. b) The structural integrity of SNT–ZnPcS_4_ (2/2 µM) after challenged by various representative biological species; Inset: Fluorescence images of ZnPcS_4_ in PBS, SNT–ZnPcS_4_ in FBS, and SNT–ZnPcS_4_ in PBS. The concentration of ZnPcS_4_ and SNT is 10 µM. c) The reduction kinetics of SNT (20 µM, 420 nm) in the presence of Na_2_S_2_O_4_ (200 mM);. d) The UV–vis spectra of SNT (4.0 µM) after incubation with NAD phosphate (NADPH) and DT‐diaphorase for 0, 5, 15, 25, 35, 45, 65, 85, and 105 min under hypoxic conditions. e) The titration curves of ZnPcS_4_ with SNT or NH_2_C4A‐12C. f) The fluorescence recovery of SNT–Fl (4/2 µM) after the addition of Na_2_S_2_O_4_ (10 µM). g) The relationships between the concentration of released PTX and the fluorescence recovery of ZnPcS_4_ from SNT–Zn/PTX (10–5/5 µM) in the presence of NADPH and DT‐diaphorase under hypoxic conditions. h) The relationships between the fluorescence recovery of ZnPcS_4_ and EY from SNT–Zn/EY in the presence of NADPH and DT‐diaphorase under hypoxic conditions. i) The relationships between the cell viability and the mean fluorescence intensity (MFI) of 4T1 cells after incubation with SNT–EY/PTX under hypoxic conditions for 24 h. The concentrations of NADPH and DT‐diaphorase used in these studies are 10 and 0.2 µM. For g‐i, Data are represented as mean ± standard deviation (s.d.) from three independent experiments (*n* = 3).

After obtaining QAAC4A‐12C, SNT was prepared by co‐assembling PEG‐12C with QAAC4A‐12C at a 1:1 molar ratio in an aqueous solution via a sonication process (more details are provided in the Supporting Information). Incorporating PEG‐12C enhances the solubility of SNT and, importantly, improves the stability of SNT in the bloodstream to prevent undesired clearance by the immune systems.^[^ ^22^ ^]^ Moreover, as the formation of SNT from the self‐assembly of QAAC4A‐12C, the amphiphilic nature of QAAC4A‐12C determines that the azo groups, which control the drug release, are located on the surface of a spherical nanoparticle. This structure ensures that all the azo groups have equal accessibility to the enzyme allowing them to be reduced synchronously by the enzyme, which sets a solid foundation for SNT to achieve real‐time tracking of drug release in vivo. The successful preparation of SNT was confirmed by dynamic light scattering (DLS) and transmission electron microscopy (TEM) measurement, resulting in the formation of unique spherical nanoparticles with a zeta potential of 9.73 ± 0.57 mV and a hydrodynamic diameter of 147.4 ± 1.6 nm (**Figure** **2**a; Figure S3, Supporting Information).

Efficient loading of drugs and fluorescence probes are the prerequisites for SNT to achieve real‐time tracking of drug release in vivo. To this end, three types of probes, including eosin Y (EY), fluorescein (Fl), and ZnPcS_4_, as well as four types of drugs, including NLG919, PTX, chlorambucil (CHL), and methotrexate (MTX), were used as guest molecules. The binding affinity (*K*_a_) of SNT to these guest molecules were determined by fluorescence titration and calculated to be (2.6 ± 0.6) × 10^7^ for EY, (2.05 ± 0.32) × 10^8^ for Fl, (5.2 ± 2.8) × 10^8^ for ZnPcS_4_, (2.38 ± 0.58) × 10^7^ for MTX, (6.21 ± 0.26) × 10^7^ for CHL, (4.81 ± 0.41) × 10^6^ for PTX, and (2.23 ± 0.67) × 10^7^ M^−1^ for NLG919, respectively^[^ ^23^ ^]^(Figure S4 and Table S1, Supporting Information). Such well‐defined binding stoichiometry and strong binding affinity ensure efficient loading of drugs and probes by SNT. For example, the loading efficiency of SNT (100 µM) can reach 98% for NLG919, 95% for PTX, and 99.5% for ZnPcS_4_ (100 µM). More importantly, this precise loading effectively quenching the photophysical properties of probes (Figure S5, Supporting Information).

As a non‐covalent supramolecular system for real‐time tracking, SNT should maintain structural integrity during blood circulation to prevent premature payload leakage and undesired fluorescence recovery of probes. For the demonstration, the fluorescence probe ZnPcS_4_ was loaded into SNT to form SNT–ZnPcS_4_, which was then challenged with major substances in the blood, including bovine serum albumin (BSA), vitamin C, glucose, etc.^[^ ^24^ ^]^ The results are summarized in Figure 2b, where no apparent fluorescence recovery of ZnPcS_4_ was observed from SNT–ZnPcS_4_ in the presence of these blood substances, suggesting the stability of SNT–ZnPcS_4_ during circulation in the bloodstream. Direct incubation of SNT–ZnPcS_4_ in FBS further confirmed this result (Figure 2b, inset). Considering the similar binding affinities between SNT for ZnPcS_4_ and the drugs/probes employed in this study, the undesired leakage of the cargoes from SNT in bloodstream can also be effectively avoided.

Next, we investigated the hypoxia responsiveness of SNT. In this study, a chemical mimic of reductase, sodium dithionite (Na_2_S_2_O_4_), was added to the SNT solution, followed by continuous monitoring of the absorbance of azophenyl at 420 nm using a UV–vis spectrometer.^[^ ^16^, ^25^ ^]^ The results are presented in Figure 2c, showing a gradual decrease in absorbance at 420 nm, which disappeared entirely within 10 min. This indicates the complete reduction of azo groups in SNT (Figure S6, Supporting Information). The mass spectra‐based analysis further validated this result, as the characteristic peak of QAAC4A‐12C at 510.3695 ([M]^4+^) disappeared, and a new peak indicating NH_2_C4A‐12C was generated at 1179.9524 [M + Na] ^+^ (Figure S7, Supporting Information). In addition, SNT could also be effectively reduced by DT‐diaphorase with NAD phosphate (NADPH) under hypoxic conditions (Figure 2d) rather than under normoxic conditions (Figure S8, Supporting Information). Subsequently, we investigated the alterations in the binding affinity of SNT to guest molecules before and after reduction by using ZnPcS_4_ as guest molecules. As shown in Figure 2e, NH_2_C4A‐12C exhibited a significantly lower (<1000 M^−1^) binding affinity to ZnPcS_4_ than QAAC4A‐12C ((5.2 ± 2.8) × 10^8^ M^−1^). This substantial reduction in binding affinity suggests that the loaded drugs can feasibly be released from SNT following the reduction. To demonstrate, Na_2_S_2_O_4_ was added into the SNT–Fl solution, and the fluorescence recovery of Fl from SNT–Fl was monitored with a microplate reader. As illustrated in Figure 2f, the fluorescence signal of Fl gradually recovered following the addition of Na_2_S_2_O_4_, suggesting the ability of SNT to achieve the controlled release of the loaded drug under hypoxic conditions (Figure S9, Supporting Information).

### 2.2 SNT for Real‐Time Tracking of Drug Release In Vitro

More importantly, the degree of fluorescence recovery of the probes effectively indicates the proportion of drugs released from SNT. For demonstration, ZnPcS_4_ and PTX were coloaded into SNT to form SNT–Zn/PTX, followed by incubation with DT‐diaphorase and NADPH under hypoxic conditions. The mixture solutions were collected at certain time points after incubation for analysis using a fluorescence spectrometer and HPLC. As shown in Figure 2g, the fluorescence intensity of ZnPcS_4_ exhibited a strong liner correlation (Pearson's r = 0.9681, *R* ^2^ = 0.9336) with the concentration of PTX released from SNT, suggesting the ability of SNT to achieve real‐time tracking of drug release. Similar results were observed from SNT–Zn/EY and SNT–EY/PTX (Figure 2h; Figures S10 and S11, Supporting Information). In addition, this real‐time tracking ability of SNT was further evaluated at the cellular level by incubating different concentrations of SNT–Zn/PTX with mouse breast cancer cells (4T1) under hypoxia conditions. After 24 h of incubation, the cells were collected for cell viability and flow cytometric analysis (Figure S12, Supporting Information). As shown in Figure 2i, the mean fluorescence intensity (MFI) of 4T1 cells is negatively correlated (Pearson's r = −0.9515, *R* ^2^ = 0.9054) with cell viability. This strong correlation could be attributed to the hypoxia‐triggered PTX release and its restoration of anti‐tumor activity. Collectively, these in vitro results suggest the great potential of SNT for real‐time tracking of drug release.

### 2.3 SNT for Real‐Time Tracking of Drug Release In Vivo

**Figure 3:**
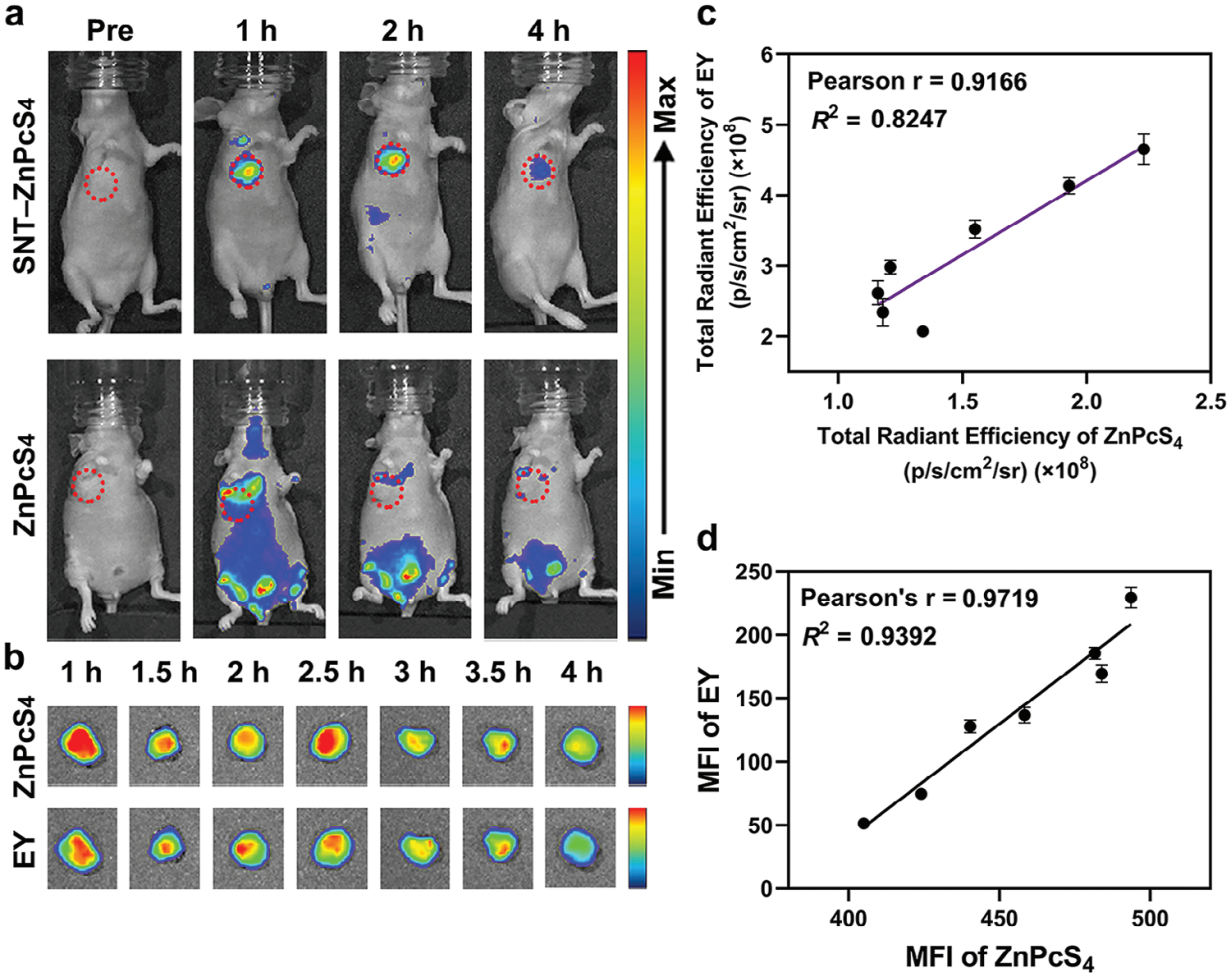
SNT for real‐time tracking of drug release in vivo. a) The biodistribution of SNT–ZnPcS_4_ and ZnPcS_4_ in 4T1‐tumor‐bearing mice. The circled red dashed line indicates tumor sites. b) *Ex vivo* imaging of tumors harvested from the SNT–Zn/EY‐treated mice at 1, 1.5, 2, 2.5, 3, 3.5, and 4 h post‐injection. c) The relationships between the total radiant efficiency of EY and ZnPcS_4_ in tumors from mice treated with SNT–Zn/EY at different time points post‐injection. d) The relationships between the MFI of EY and ZnPcS_4_ in tumors from mice treated with SNT–Zn/EY at different time points post‐injection. Data are represented as mean ± s.d. from three independent experiments (*n* = 3).

To achieve real‐time tracking of drug release in vivo, SNT should efficiently co‐deliver the loaded probes and drugs to tumor tissues. To demonstrate, SNT–ZnPcS_4_ was intravenously injected into 4T1 tumor‐bearing BALB/c nude mice, with free ZnPcS_4_ used as a negative control. As the results summarized in **Figure** **3**a, the fluorescence in mice treated with SNT–ZnPcS_4_ was predominantly localized at the tumor site, peaking at 2 h post‐injection, suggesting that SNT released the majority of ZnPcS_4_ specifically within the tumor tissues. In contrast, the free ZnPcS_4_ treated group showed no obvious on‐target distribution. More importantly, SNTs are required to release the loaded drugs and probes simultaneously upon reaching tumor tissues. For demonstration, two probes, EY and ZnPcS_4_, were coloaded into SNT (SNT–Zn/EY) and then intravenously injected into 4T1 tumor‐bearing mice. The mice were sacrificed at predetermined time points post‐injection, and their tumor tissues were collected for IVIS and flow cytometric analysis. As demonstrated in Figure 3b,c, the total radiant efficiency of EY and ZnPcS_4_ in tumor tissues showed a highly positive correlation (Pearson's r = 0.9166, *R* ^2^ = 0.8247). This suggests that EY and ZnPcS_4_ were simultaneously released from SNT and underwent a similar metabolic behavior in tumor tissues. Flow cytometry‐based analysis further confirmed these results (Figure 3d), in which the MFI of EY exhibited a strong linear correlation (Pearson's r = 0.9719, *R* ^2^ = 0.9392) with the MFI of ZnPcS_4_. These findings indicate the ability of SNT to co‐deliver the loaded drugs and probes to tumor tissues and release them synchronously. Different from conventional real‐time tracking systems that rely on developing photocaged compound or utilizing FRET effect, SNT achieves to load drugs and probes through host‐guest interaction, effectively avoiding complicated synthesis processes. Additionally, the rationally designed chemical structure of QAAC4A‐12C enables SNT to release them synchronously in the hypoxic TME, making SNT an ideal platform for real‐time tracking of drug release in vivo.

In addition, we also investigated the tumor‐targeted delivery efficiency of SNT by employing 4‐(9,9‐Dioctylfluoren‐2‐yl)−2,1,3‐benzothiadiazole (F8BT) as the fluorescence probe. In details, F8BT was first co‐assembled with QAAC4A‐12C and PEG‐12C to form SNT@F8BT that emit fluorescence under both normoxic and hypoxic conditions. Subsequently, SNT@F8BT was intravenously injected into 4T1‐bearing BALB/c mice. At 1, 2, 4, and 6 h post‐injection, the mice were sacrificed, and the tumors and major organs were collected for ex vivo observation. As shown in Figure S13, fluorescence was predominantly localized in the liver, kidneys, intestines, and tumors. Further quantitative analysis based on the fluorescence intensity from ex vivo images indicated that ≈7.88%, 7.93%, 6.87% and 4.47% of SNT accumulated in tumor tissues at 1‐, 2‐, 4‐ and 6 h post‐injection, respectively.

### 2.4 SNT‐Zn/PTX for Chemo‐Photodynamic Combination Therapy

**Figure 4:**
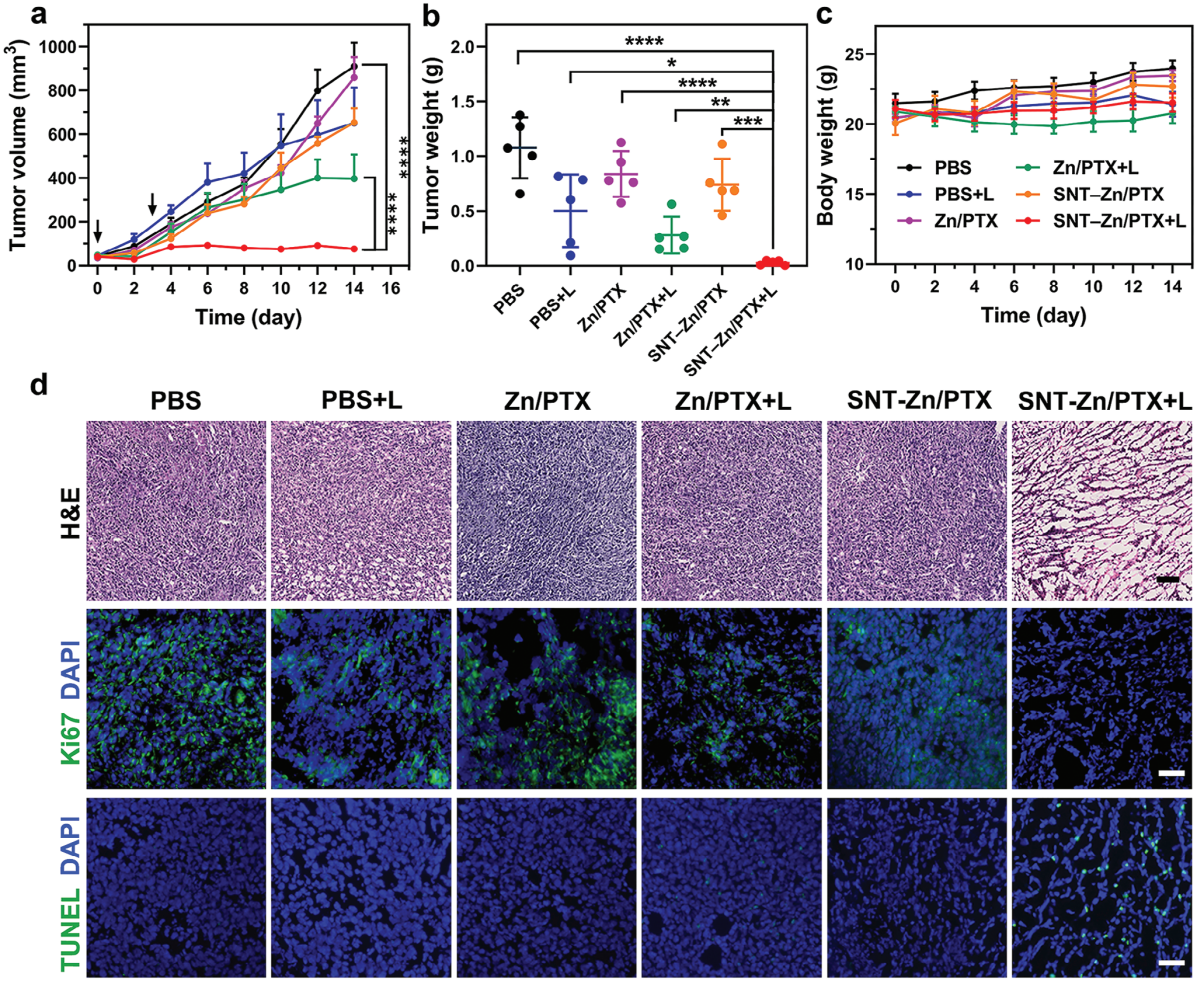
SNT for effective chemo‐photodynamic combination therapy. a) The growth kinetics of the tumor after receiving different treatments (PBS, PBS+L, Zn/PTX, Zn/PTX+L, SNT–Zn/PTX, and SNT–Zn/PTX+L). b) The tumor weight in each group at the end of the experiment. c) The changes in body weight during the treatment. d) The tumor sections in each group were stained with H&E, Ki67, and TUNEL. The scale bars are 100 µm. Data are represented as mean ± s.d. (*n* = 5), and the significance levels are **p < 0.01, ***p < 0.001, and ****p < 0.0001, analyzed by two‐way ANOVA for (a), T‐test for (b).

In addition to real‐time tracking of drug release in vivo, the released drugs may synergize effectively with fluorescent probes upon light irradiation, achieving a potent chemo‐photodynamic combination therapy for tumors. To this end, ZnPcS_4_ and PTX were employed as model combinations and coloaded into SNT to form SNT–Zn/PTX, which was then administrated into 4T1‐bearing mice (*n* = 5). At 2 h post‐injection, the tumors were exposed to 660 nm laser irradiation for 3 min (denoted as SNT–Zn/PTX+L, where L represents 660 nm irradiation). The irradiation window was determined by its maximum accumulation in tumor tissues (Figure 3a). Mice receiving PBS, PBS+L, the mixture of ZnPcS_4_ and PTX (denoted as Zn/PTX), Zn/PTX+L, and SNT–Zn/PTX were used as control groups. As illustrated in **Figure** **4**a, only slightly tumor suppressions were observed in PBS+L, Zn/PTX, Zn/PTX+L, and SNT–Zn/PTX‐treated mice than that of PBS. In contrast, SNT–Zn/PTX+L treatment resulted in significantly enhanced anti‐tumor efficacy. These results were further confirmed by directly observing the tumor tissues (Figure S14, Supporting Information) and comparing tumor weight (Figure 4b), which revealed that mice treated with SNT–Zn/PTX+L had the smallest and lightest tumor tissues. In addition, no obvious variations in body weight were observed during treatment (Figure 4c). Further analysis, including blood routine, serum enzyme assays, and histopathological (Figure S15, Supporting Information), revealed that no significant organ damage or abnormalities were detected in mice treated with SNT‐based formulations. On day 14, mice were sacrificed, and the tumors were collected and sliced for further analysis. As illustrated in Figure 4d, the tumor sections from SNT–Zn/PTX+L treated mice exhibited obvious apoptotic features, including the exodus of nuclei, as well as hyperchromatic and condensed nuclei. Immunofluorescence staining‐based analysis further confirmed these results, with sections from mice treated with SNT–Zn/PTX+L showing significantly decreased proliferative signals (Ki67, Figure 4d, middle) and enhanced apoptotic signals (TUNEL, Figure 4d, bottom), indicating the great potential of SNT for enhanced chemo‐photodynamic combination therapy.

### 2.5 SNT‐Zn/NLG919+L for Enhanced Cancer Immunotherapy

**Figure 5:**
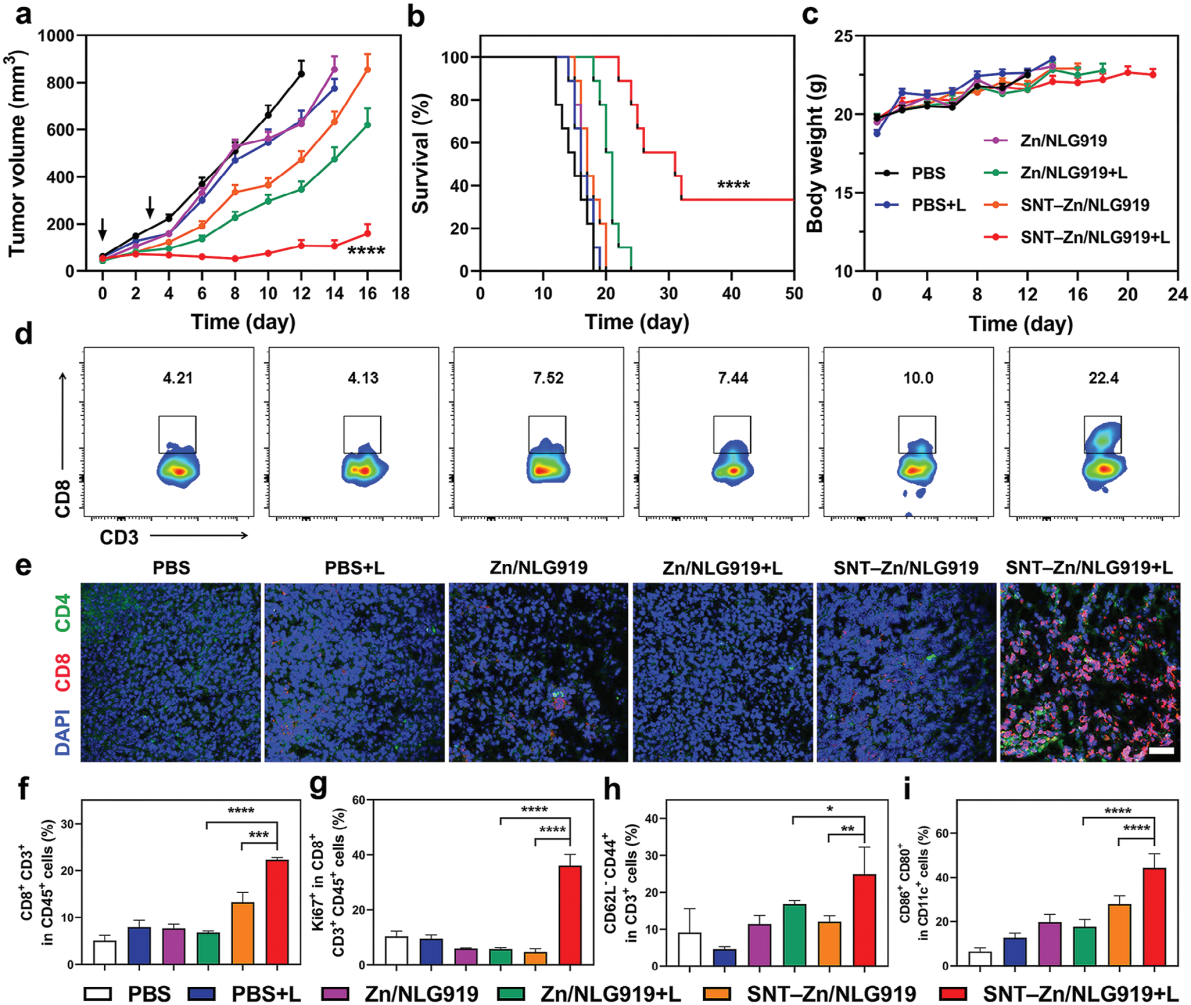
SNT for effective cancer immunotherapy. a) The growth kinetics of the tumor after receiving different treatments (PBS, PBS+L, Zn/NLG919, Zn/NLG919+L, SNT–Zn/NLG919 and SNT–Zn/NLG919+L). b) The survival rate of tumor‐bearing mice after receiving different treatments. c) The changes in body weight during the treatment. d) Flow cytometric analysis of the infiltration of CD8^+^ T cells in tumors. e) Representative immunofluorescence images of tumors showing CD4^+^ T cell and CD8^+^ T cell infiltration from the mice. The scale bar is 50 µm. f–i) Quantitative analysis of the infiltration of CD8^+^ T cells (f, gated on CD45^+^CD3^+^ cells), proliferation T cells (g, gated on CD45^+^CD3^+^Ki67^+^), T effector memory cells (h, gated on CD45^+^CD3^+^CD62^−^CD44^+^), the maturation of DC cells (i, gated on CD11c^+^CD80^+^CD86^+^cells) in each group according to corresponding flow cytometric analysis. Data are represented as mean ± s.d. *n* = 9 for a‐c, and *n* = 4 for **f–i**. The significance levels are *p < 0.05, **p < 0.01, ***p < 0.001, ****p < 0.0001, analyzed by two‐way ANOVA for **a** and T‐test for f‐i.

Recent studies revealed that PDT‐treated cancer cells undergo a unique pattern of cell death, namely immunogenic cell death (ICD).^[^ ^26^ ^]^ During ICD, the dying cancer cells function similarly to in‐situ cancer vaccines, thus inducing a systemic antitumor immune response.^[^ ^27^ ^]^ However, the immunity induced by ICD also triggers the upregulation of immunosuppressive factors, for example, indoleamine 2,3‐dioxygenase 1 (IDO‐1), which limits the survival and function of CD8^+^ T cells.^[^ ^28^ ^]^ Therefore, using various modulators or drugs to simultaneously regulate multiple immune pathways, such as combining IDO‐1 inhibition with ICD activation, has become a promising strategy for enhancing the tumor suppression efficacy of cancer immunotherapy.^[^ ^29^ ^]^ To demonstrate, NLG919 (an IDO‐1 inhibitor) and ZnPcS_4_ were employed as drug combinations and co‐loaded into SNT (denoted as SNT–Zn/NLG919) to explore its potential for enhanced cancer immunotherapy. In detail, 4T1 tumor‐bearing BALB/c mice were randomized into three groups (*n* = 18), followed by intravenous injection of PBS, ZnPcS_4_ and NLG919 mixture (denoted as Zn/NLG919), and SNT–Zn/NLG919. Two hours post‐injection, the mice were further divided into two subgroups, with half of the mice receiving irradiation (denoted as + L) and the other half not. As shown in **Figure** **5**a, SNT–Zn/NLG919+L treatment resulted in significantly enhanced tumor suppressions than all other interventions, indicating the optimal synergistic anti‐tumor efficacy of SNT–Zn/NLG919+L. As a result, SNT–Zn/NLG919+L greatly prolonged the survival curve of 4T1‐bearing mice, with 33.33% still alive within the observed 50 days (Figure 5b; Figure S16, Supporting Information). Furthermore, no obvious body weight variations were observed during treatment (Figure 5c).

Next, we investigated the potential mechanism underlying this significantly enhanced anti‐tumor efficacy. Tumors from each group were collected and homogenized for flow cytometry analysis on day 7 after irradiation. As illustrated in Figure 5d, negligible increases in CD8^+^ tumor‐infiltrating lymphocytes (TILs, in CD45^+^CD3^+^ cells) were detected from tumors in PBS+L (4.13%), Zn/NLG919 (7.52%), and Zn/NLG919+L (7.44%)‐treated mice compared to the PBS group. Meanwhile, only a slightly enhanced infiltration of CD8^+^ TILs was observed in SNT–Zn/NLG919‐treated mice (10.0%). Noteworthy, CD8^+^ TILs are significantly increased in SNT–Zn/NLG919+L‐treated mice (22.4%) by more than 5‐fold compared to the PBS group and by 3.0 and 2.24‐fold compared to the Zn/NLG919+L and SNT–Zn/NLG919‐treated mice respectively, indicating the significantly enhanced CD8^+^ TILs infiltration induced by SNT–Zn/NLG919+L.^[^ ^30^ ^]^ These results were further confirmed by immunofluorescence staining, in which a significant higher red fluorescence signal indicating CD8^+^ T cells was observed from tumor sections in SNT–Zn/NLG919+L treated mice (Figure 5e). In addition, the infiltrated CD8^+^ TILs in SNT–Zn/NLG919+L‐treated mice are highly proliferative (23.6%, Figure 5f) compared to other interventions. Moreover, SNT–Zn/NLG919+L also induced systemic antitumor immunity, as confirmed by significantly enhanced CD8^+^ effector memory T cells (31.8%, in CD44^+^CD62L^−^) in spleen and dendritic cell (DC) maturation ratio (53.8%, in CD11b^+^) in lymph node (Figure 5h,i; Figures S17, S18 and S19, Supporting Information). In summary, these findings indicate that SNT‐Zn/NLG919+L effectively activates ICD‐associated anti‐tumor immunity, thereby significantly bolstering tumor suppression.

## 3 Conclusion

In summary, we have developed SNT capable of precise loading and tumor‐targeted delivery of drugs and fluorescence probes, enabling real‐time tracking of drug release and effective chemo‐photodynamic combination therapy. SNT is primarily composed of QAAC4A‐12C, a hypoxia‐responsive supramolecular receptor with a strong binding affinity toward various drugs and fluorescence probes. By integrating multiple cavities into a single ensemble, SNT achieves co‐loading of drugs and probes while efficiently quenching the photophysical properties of the probe through complexation under physiological conditions. Upon reaching hypoxic TME, QAAC4A‐12C gets effectively reduced, leading to the simultaneous release of drugs and probes and the fluorescence recovery of probes. With this spatial and temporal consistency in drug loading and fluorescence quenching, as well as drug release and fluorescence recovery, SNT successfully achieves real‐time tracking of drug release in vivo. Moreover, the released drugs synergize effectively with fluorescent probes upon light irradiation, achieving potent chemo‐photodynamic combination therapy for tumors. Considering that the lack of actual drug concentrations released from nanoDDS is a major limiting factor for their widespread clinical application, we foresee that real‐time tracking platforms like SNT will greatly advance the development of nanomedicine and are expected to achieve optimal therapeutic effects in the clinic.

## Conflict of Interest

The authors declare no conflict of interest.

## Supporting information

Supporting Information

## Data Availability

Research data are not shared.
