# Supplementary material for: Supramolecular Nano‐Tracker for Real‐Time Tracking of Drug Release and Efficient Combination Therapy
Source: Adv Sci (Weinh). 2024 Jul 28;11(36):2404731. doi: 10.1002/advs.202404731 (PMC11423228; doi:10.1002/advs.202404731)
Supplement: Supplementary file 1 — Supporting Information [file ADVS-11-2404731-s001.docx]

Supramolecular Nano-tracker for Real-time Tracking of Drug Release and Efficient Combination Therapy

Xi Chen, Fang-Yuan Chen, Yi Lu, Qiushi Li, Shujie Li, Chunxiong Zheng, Yadan Zheng, Lin Dang, Ru-Yi Li, Yang Liu*, Dong-Sheng Guo*, Shao-Kai Sun*, and Zhanzhan Zhang*

X. Chen, Y. Lu, S. J. Li, Prof. S.-K. Sun, Dr. Z.Z. Zhang

School of Medical Imaging, Tianjin Key Laboratory of Functional Imaging, Tianjin Medical University, Tianjin 300203, China

E-mail: shaokaisun@tmu.edu.cn; [zzz@tmu.edu.cn](mailto:zzz@tmu.edu.cn)

F.-Y. Chen, Q. S. Li, Ru-Yi Li, Prof. Y. Liu, Prof. D.-S. Guo

College of Chemistry, Key Laboratory of Functional Polymer Materials (Ministry of Education) State Key Laboratory of Medicinal Chemical Biology, Nankai University, Tianjin 300071, China

E-mail: yliu@nankai.edu.cn; dshguo@nankai.edu.cn

Dr. C. X. Zheng

School of Chemistry, South China Normal University, Guangzhou, 510006, China.

Dr. Y. D. Zheng

College of Veterinary Medicine, Northeast Agricultural University, Harbin 150030, PR China

Dr. L. Dang

Precision Medicine Center, Tianjin Medical University General Hospital, Tianjin 300000, China

**Table of contents**

[1.1 Materials 3](#_Toc171691128)

[1.2 Instruments 3](#_Toc171691129)

[1.3 Cell culture 4](#_Toc171691130)

[1.4 Preparation and characterization of SNT 4](#_Toc171691131)

[1.5 Data analysis of fluorescence titrations 4](#_Toc171691132)

[1.6 Fluorescence responses of SNT–ZnPcS_4_ upon the addition of various biologically coexisting species 6](#_Toc171691133)

[1.7 Study of the hypoxia-responsive ability of QAAC4A-12C 6](#_Toc171691134)

[1.8 Study of the real-time tracking ability of SNT *in vitro* 6](#_Toc171691135)

[1.9 In vivo fluorescence imaging 7](#_Toc171691136)

[1.10 Analysis of the anti-tumor efficacy of SNT–Zn/PTX 7](#_Toc171691137)

[1.11 Biosafety evaluation 8](#_Toc171691138)

[1.12 Analysis of the anti-tumor efficacy of SNT–Zn/NLG919 8](#_Toc171691139)

[1.13 Immunofluorescence staining 8](#_Toc171691140)

[1.14 Flow cytometry analysis 8](#_Toc171691141)

[1.15 Statistical Analysis 9](#_Toc171691142)

[2. Syntheses of QAAC4A-12C 10](#_Toc171691143)

[3. Supporting results and experimental data 11](#_Toc171691144)

[3.1 Characterization of SNT 11](#_Toc171691145)

[3.2 Binding affinities of SNT with guests 11](#_Toc171691146)

[3.3 Hypoxia responsiveness of SNT–ZnPcS_4_ 12](#_Toc171691147)

[3.4 Synchronous release of the cargo through SNT 15](#_Toc171691148)

[3.5 The biodistribution of SNT@F8BT 16](#_Toc171691149)

[3.6 Individual tumor growth kinetics in different groups 17](#_Toc171691150)

[3.7 Biosafety analysis of SNT–Zn/PTX 17](#_Toc171691151)

[3.8 Individual tumor growth kinetics in different groups 18](#_Toc171691152)

[3.9 Flow cytometry analysis of cells 18](#_Toc171691153)

[Table S1. Binding affinities of QAAC4A-12C and NH_2_C4A-12C with guest molecules. 19](#_Toc171691154)

[Reference: 19](#_Toc171691155)

1. General methods and materials

## **1.1 Materials**

All the reagents and solvents that were commercially available were used as received without further purification unless otherwise noted. Paclitaxel (PTX) and bovine serum albumin (BSA) were purchased from Shanghai Aladdin Chemistry Co., Ltd (Shanghai, China). DT-diaphorase were purchased from Shanghai Sangon Biotech Co., Ltd (Shanghai, China). Hematoxylin-Eosin/H&E staining kit, Epirubicin HCl (EPI), Daunorubicin HCl (DNR), and Mitomycin C (MMC) were purchased from Beijing Solarbio Science & Technology Co., Ltd. Sodium dithionite (Na_2_S_2_O_4_) was purchased from Across. Phosphate Buffered Saline (PBS) was purchased from Procell Life Science&Technology Co., Ltd. Nicotinamide adenine dinucleotide phosphate (NADPH), terminal deoxynucleotidyl transferase dUTP nick end labeling (TUNEL) kit and Ki67 kit were purchased from Beyotime Biotechnology (Shanghai, China). Alanine was purchased from TCI. Valine was purchased from Across. Glycine, glucose, creatinine was purchased from J&K. Lysine was obtained from Meryer. Sodium dihydrogen phosphate was purchased from 3A chemistry. Sodium chloride was purchased from Benchmark. Glutathione was purchased from Energy Chemical. Glycidyltri methylammonium chloride was purchased from Adamas. Sulfonated zinc phthalocyanine (ZnPcS_4_) was obtained from Bide Pharmatech Co., Ltd. Antibodies for confocal laser scanning microscope observation (CLSM) were purchased from Biolegend, Beyotime Biotechnology (Shanghai, China) and Proteintech as follow-CD4 primary antibody (anti-CD4 from Rabbit, Proteintech), CD8 primary antibody (anti-CD8 from mouse, Proteintech), FITC-labeled Goat Anti-Rabbit IgG (H+L) (Beyotime), Alexa Fluor 647-labeled Goat Anti-Mouse IgG(H+L) (Beyotime). Antibodies for flow cytometry were obtained from Biolegend. 4-(Dodecyloxy)benzamido-terminated methoxy poly(ethylene glycol) (PEG-12C) was synthesized according to the previous literature^[1]^.

## **1.2 Instruments**

NMR data were recorded on a AVANCE Ⅲ spectrometer. UV–vis spectra were recorded in a UV–vis spectrophotometer (Shimadzu, Japan). The fluorescence emission spectra were measured on traditional (F7000, Hitachi, Japan) fluorescence spectrometers. Mass spectra were performed on a Waters Xevo G2-XS Q-Tof mass spectrometer. The size distribution and zeta potential studies were performed on a Malvern Zetasizer (Nano series ZS, UK). Transmission Electron Microscopy (TEM) measurements were performed on a Talos F200C electron microscope at an acceleration voltage of 120 kV. Cell viability was measured on a BioTEK, USA. CLSM images were captured on a FluoView Confocal Laser Scanning Microscopes-FV1000. Living imaging was performed with an IVIS Lumina imaging system (Caliper Life Sciences, USA). High Performance Liquid Chromatography (HPLC) analysis was carried on Waters Alliance e2695 with a Symmetry C_18_ Column (5 μm, 4.6 m × 150 mm) at 25 ˚C.

## **1.3 Cell culture**

The mouse breast cancer cells 4T1 were purchased from the American Type Culture Collection. 4T1 cells were maintained in RPMI 1640 medium with 10 % fetal bovine serum (v/v), 100 U/ml penicillin and streptomycin. For the normoxia condition, the tumor cells were incubated in a humidified atmosphere of 5 % CO_2_ at 37 ˚C. For the hypoxia condition, the tumor cells were incubated in a humidified atmosphere of 94 % N_2_, 5 % CO_2_, 1 % O_2_ at 37 ˚C。

## **1.4** **Preparation and characterization of SNT**

6 mg of QAAC4A-12C and 5.8 mg of PEG-12C (molar ratio 1 : 1) were dissolved in 8.4 mL of HEPES buffer (10 mM, pH 7.4) and then mixed fully by sonicating at 80 ˚C for 30 min to obtain SNT.

For the preparation of TEM sample, SNT was first diluted to 10 μM, followed by the drop-coating of 2 μL SNT onto carbon-coated copper grids (Beijing Zhongjingkeyi Technology Co., Ltd, China). Droplet of sample was contacted with the grids for 5~10 min, then excess amount of sample was removed. The grid was then rinsed and stained with 1 % phosphotungstic acid hydrate (5~10 μL) for 1.5 min, and then observed with a transmission electron microscopy (TEM, FEI Talos F200C electron microscope).

## **1.5** **Data analysis of fluorescence titrations**

The fitting of data from direct host-guest titrations was performed in a nonlinear manner^[2]^, and the fitting modules were downloaded from the website of Prof. Nau’s group (http://www.jacobs-university.de/ses/wnau) under the column of “Fitting Functions”. It was introduced in detail as following:

For analyzing the host-guest direct titrations (equation **1**), we considered that a guest (G) formed a 1:1 host–guest complex with a host (H) at an association constant (*K*_a_), which satisfied the respective law of mass action relating to the equilibrium concentrations of free host, [H], free guest, [G], and host–guest complex [HG]. Also, the relationship between the total concentrations of host, [H]_0_, and guest, [G]_0_, and their equilibrium concentrations were introduced by the law of mass conservation (equation **2**). Here, [G]_0_ was the initial concentration of guest as a known experimental parameter, which was kept constant in the titration process. Furthermore, equation **1** and **2-1** were employed to deduce equation **3**.

When the fluorescence titrations were performed, the intensity of fluorescence (F) corresponded to the combined intensity of the guest and the host–guest complex, which were described by their molar fractions (equation **4**). Both FHG and FG were the known experimental parameters, in which FHG was the fluorescence intensity when all guests were complexed and FG when they were not complexed. The equation **5** deduced by equation **2-2**, **3** and **4**, explained the relationship between *K*_a_ and variables ([H]_0_) in fluorescence titrations. In the light of equation **5**, *K*_a_ was obtained by fitting the data of fluorescence intensity and total host concentration.


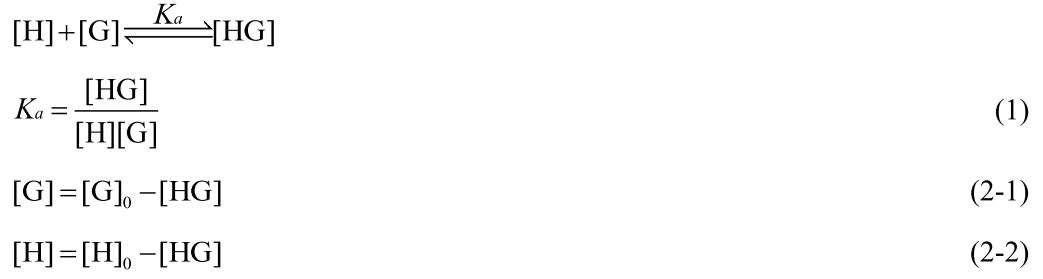

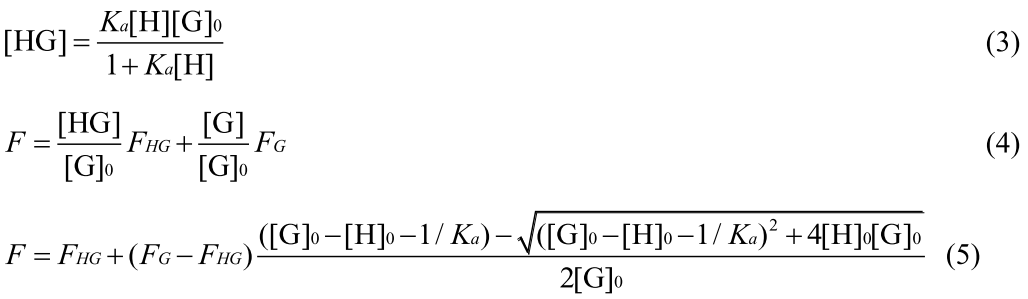


For the analysis of the competitive titrations (equation **6**), we also considered a competitor that could bind to a host’s cavity in a 1:1 stoichiometry at an association constant (*K*_c_). Free host, [H], free competitor, [C], and host–competitor complex [HC] obeyed the respective law of mass action referring to the equilibrium concentrations. Also, [H]_0_ and the total concentrations of H, [C]_0_, and their equilibrium concentrations satisfied the law of mass conservation (equation **7**).

In the course of the titration, the fluorescence intensity (FC) was expressed as a linear combination of FHG and FG, weighted by their molar fractions on the basis of equation **8**. Through a 1:1 host–guest binding model, FHG was further denoted by the (initial) experimental fluorescence intensity in the absence of competitor. Substituting equation **3** into equation **8** gave equation **9**, with the concentration of free host as an unknown parameter, [H], which was numerically solved by a cubic equation (equation **10**)^[3]^. In addition, equation **10** was deduced by combining equation **3**, **6**, **7-1** and **7-2**. For fitting, the fluorescence intensity was plotted against [C]0 based on equation **9** in a program.


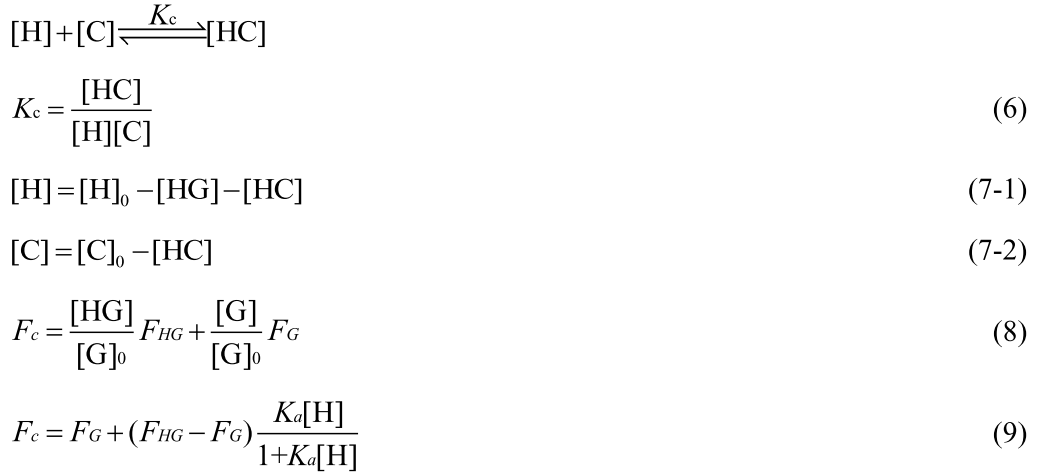

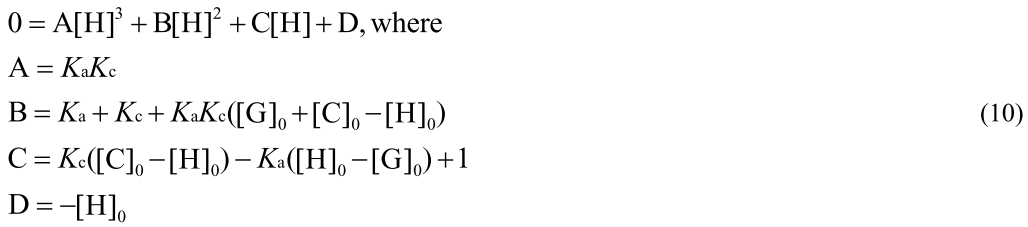


## **1.6** **Fluorescence responses of SNT–ZnPcS_4_ upon the addition of various biologically coexisting species**

Various biological coexisting species of blood were added separately to SNT–ZnPcS_4_ (2/2 μM) in HEPES (10 mM, pH 7.4) at 25 ˚C and stirred for 30 min to monitor the fluorescence intensity of ZnPcS_4_. The fluorescence of ZnPcS_4_ alone (2 μM) was used as control. The biological coexisting species and their concertation used in these experiments were: bovine serum albumin (BSA) 10 µg/mL, phosphate 5 mM, chloride 4.5 mM, vitamin C 0.1 mM, glucose 5.0 mM, Urea 6.0 mM, glutathione 300 μg/L, serine 0.2 mM, proline 0.05 mM, cysteine 0.01 mM, valine 0.2 mM, lysine 0.07 mM, glutamine 0.5 mM, alanine 0.5 mM and arginine 0.01 mM. The concentrations of all above components refer to their concentrations in human blood^[4]^.

## **1.7** **Study of the hypoxia-responsive ability of QAAC4A-12C**

The hypoxia responsiveness of QAAC4A-12C was investigated using UV–vis spectroscopy. Briefly, sodium hydrosulfite (Na_2_S_2_O_4_, 2 mM) was added into SNT (10 μM) solution in HEPES (10 mM, pH 7.4), and the absorbance of SNT at 420 nm was continuously monitored for 20 min. In addition to Na_2_S_2_O_4_, the time-dependent absorbance of SNT (10 μM) was also investigated in the presence of nicotinamide adenine dinucleotide phosphate (NADPH, 10 μM) and DT-diaphorase (0.2 μM) at 25 ˚C. Note: Argon gas was bubbled into the solution to create the hypoxic environment. The hypoxia-triggered drug release of SNT–ZnPcS_4_ was also investigated.

## **1.8** **Study of the real-time tracking ability of SNT *in vitro***

In this study, ZnPcS_4_ and PTX were first loaded into SNT to form SNT–Zn/PTX (SNT 5 μM, ZnPcS_4_: PTX=1:1, 2.5 μM/2.5 μM), followed by incubation with NADPH (10 μM) and DT-diaphorase (0.2 μM) under hypoxic conditions. Note: Argon gas was bubbled into the solution to create the hypoxic environment. The mixture solutions were collected at certain timepoints after incubation for analysis. The fluorescence recovery of ZnPcS_4_ from SNT was recorded via fluorescence spectrophotometer, while the released PTX from SNT was detected by HPLC. Similar study was performed on SNT–Zn/EY by replacing PTX with EY.

In addition, the real-time tracking ability of SNT was further evaluated at the cellular level by incubating different concentration of SNT–EY/PTX (EY: PTX=1:1, ranging from 0.234 μM to 15 μM) with mouse breast cancer cells (4T1) under hypoxia conditions for 24 hours. After incubation, the cells were collected for cell viability (CCK-8 assay according to manufacturer’s protocol) and flow cytometric analysis.

## **1.9** **In vivo fluorescence imaging**

All animal procedures were performed in accordance with the Guidelines for Care and Use of Laboratory Animals of Tianjin Medical University and experiments were approved by the Animal Ethics Committee of Tianjin Medical University (approve number, IACUC 23JCQNJC00280).

In this study, female BALB/c mice and female nude BALB/c nude mice at 6-8 weeks were purchased from SPF (Beijing) Biotechnology Co., Ltd. To establish the xenograft 4T1 tumor-bearing mouse model, 1×10^6^ 4T1 cancer cells were injected subcutaneously into the right mammary fat pad of BALB/c nude mice. To investigate the tumor-targeted delivery ability of SNT, the BALB/c nude mice with tumor volumes at around 100 mm^3^ were randomized into two groups and intravenous injected with 200 μL of ZnPcS_4_ and SNT–ZnPcS_4_ with a dosage of 1.79 mg^-1^. The *in vivo* biodistribution of ZnPcS_4_ were collected at 0, 1, 2, and 4 h post injection. To investigate the real-time tracking ability of SNT in vivo, SNT–Zn/EY was intravenously injected into 4T1-bearing BALB/c mice (tumor volumes at around 100 mm^3^), the mice were sacrificed at 1, 1.5, 2, 2.5, 3, 3.5, and 4 h post-injection, and the tumor were collected for *ex vivo* imaging. Fluorescent images were analyzed using an IVIS Lumina imaging system (Caliper Life Sciences, USA) and analyzed using Living Image 3.1 (Caliper Life Sciences).

To investigate the biodistribution of SNT *in vivo*, we applied 4-(9,9-Dioctylfluoren-2-yl)-2,1,3-benzothiadiazole (F8BT) as the fluorescence probe, co-assembled with QAAC4A-12C and PEG-12C to form SNT@F8BT. This co-assembly process effectively retains the photophysical properties of F8BT, allowing SNT@F8BT to emit fluorescence even under non-hypoxic conditions. Subsequently, SNT@F8BT was intravenously injected into 4T1 tumor-bearing BALB/c nude mice. At 1, 2, 4 and 6 h post-injection, the mice were sacrificed, and the major organs were collected for *ex vivo* observation.

## **1.10** **Analysis of the anti-tumor efficacy of SNT**–**Zn/PTX**

To investigate the anti-tumor efficacy of the combination of ZnPcS_4_ and PTX female BALB/c mice were subcutaneous injected with 1×10^6^ 4T1 cancer cells at the right mammary fat pad. The mice with tumor volumes at around 100 mm^3^ were randomized into three groups (*n* = 10) and intravenous injected with 200 μL of PBS, the mixture of ZnPcS_4_ and PTX (Zn/PTX, ZnPcS_4_/PTX = 200 μM/200 μM), and SNT–Zn/PTX (400 μM, ZnPcS_4_/PTX = 200 μM/200 μM) for twice on day 0 and day 3. Two hours post-injection, the mice were further divided into two subgroups, with half of the mice receiving irradiation (denoted as + L) and another half not. The tumor volume was measured by a Vernier caliper and the volume (V) was calculated by using the formula:


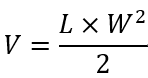


(L, the longest dimension; W, the shortest dimension)

To assess potential toxicities, mice were monitored for weight loss and euthanized when exhibiting signs of impaired health.

## **1.11** **Biosafety evaluation**

Histopathological assessment, blood biochemical analysis, and blood routine analysis were performed to evaluate the *in vivo* toxicity of SNT–Zn/PTX. In details, female BALB/c mice (6-8 weeks, 18-20 g) were randomized into two groups (*n* = 3), followed by intravenously injected with 200 μL of SNT–Zn/PTX (400 μM, ZnPcS_4_/PTX = 200 μM/200 μM) and PBS. After 14 days post-injection, the mice were euthanatized to harvest major organs for hematoxylin and eosin (H&E) staining. Meanwhile, the blood samples of mice were collected for serum biochemistry and hematological analysis.

## **1.12** **Analysis of the anti-tumor efficacy of SNT**–**Zn/NLG919**

To investigate the anti-tumor efficacy of the combination of ZnPcS_4_ and NLG919, female BALB/c mice were subcutaneous injected with 1×10^6^ 4T1 cancer cells at the right mammary fat pad. The mice with tumor volumes at around 100 mm^3^ were randomized into three groups (*n* = 18) and intravenous injected with 200 μL of PBS, the mixture of ZnPcS_4_ and NLG919 (Zn/NLG919, ZnPcS_4_/NLG919 = 150 μM/150 μM), and SNT–Zn/PTX (300 μM, ZnPcS_4_/NLG919 = 150 μM/150 μM) for twice on day 0 and day 3. Two hours post-injection, the mice were further divided into two subgroups, with half of the mice receiving irradiation (denoted as + L) and another half not. The tumor volume was measured by a Vernier caliper and the volume (V) was similarly calculated by using the abovementioned formula. To assess potential toxicities, mice were monitored for weight loss. Animals were euthanized when exhibiting signs of impaired health or when the volume of the tumor exceeded 1cm^3^.

## **1.13** **Immunofluorescence staining**

Immunofluorescence staining was performed on frozen tumor sections. Briefly, harvested tumors were first placed in 4 % paraformaldehyde for 48 h at 4 ˚C, and then transferred to 15 % then 30 % sucrose solution (w/w) for dehydration. The tumors were embedded in optimal cutting medium, and frozen slices were made on a cryostat microtome. Immunofluorescence staining was performed by rinsing with PBS, permeabilization, followed by blocking in 5 % BSA at room temperature for 30 min, and then stained with different primary antibodies including CD4 (anti-CD4 from Rabbit) and CD8a (anti-CD8a from Rat) overnight at 4 ˚C according to the manufacturer’s instructions. Following by the addition of fluorescently labelled secondary antibodies, the slides were analyzed with a fluorescent inverted microscope. All antibodies used in the experiments were diluted 200 times.

## **1.14** **Flow cytometry analysis**

Freshly harvested tumor tissues were cut into small pieces and homogenized with a glass homogenizer followed by passing through a 70 × 10^−6^ m cell strainer for single-cell suspensions. Subsequentially, the cells were collected and diluted to 1×10^7^ cells/mL. 100 μL cells were stained by adding a cocktail of fluorescent conjugated antibodies. For intracellular staining, cells were firstly permeabilized with 100 μL fixation/permeabilization buffer, and then stained with the antibody cocktail. After the staining, the cells were fixed with 4 % paraformaldehyde and analyzed via flow cytometer.

## **1.15** **Statistical Analysis**

Data represent mean ± standard deviation (s.d.) from at least three independent experiments (*n* ≥ 3) and the significance levels are **P*<0.05, ***P*<0.01, ****P*<0.001 and *****P*<0.0001, analyzed by T test or two-way ANOVA (when more than two groups were compared) with a Dunnett test. *P* < 0.05 or less was considered significant.

# **2. Syntheses of QAAC4A-12C**

**Figure S1**. The synthetic route of QAAC4A-12C

For the synthesis of QAAC4A-12C (**3**), aminocalix[4]arene pentadodecyl ether (NH_2_C4A-12C, **1**) was first synthesized according to the previous literature^[5]^. To a solution of **1** (0.9 g, 0.78 mmol) in THF (10 mL), HCl (1 M, 9 mL) and NaNO_2_ (0.38 g, 5.5 mmol, 7mL in H_2_O) were added in sequence at 0 ˚C. After stirring at room temperature for 1 h, 0.2 g phenol (2.12 mmol) in pyridine (18 mL) was added drop wisely into the solution mixtures and stirred at room temperature for another 16 h. The **2** was precipitated by dropping the reaction solution into 200 mL H_2_O, collected by vacuum filtration, and then purified by column chromatography (DCM MeOH = 40:1) (yield, 240 mg, 20%).

To a solution of **2** (240 mg, 0.15 mmol) in isopropanol (20 mL), glycidyltrimethylammonium chloride (1.38 g, 9 mmol) was added. The mixture was refluxed for 18 h and stirred at room temperature for another 2 h. Yellow precipitate was obtained (if it is still clear liquid, stir in ice water bath for 10 min). The precipitate was then filtered in vacuum and rinsed with isopropyl alcohol to obtain **3** (265 mg, 80%).


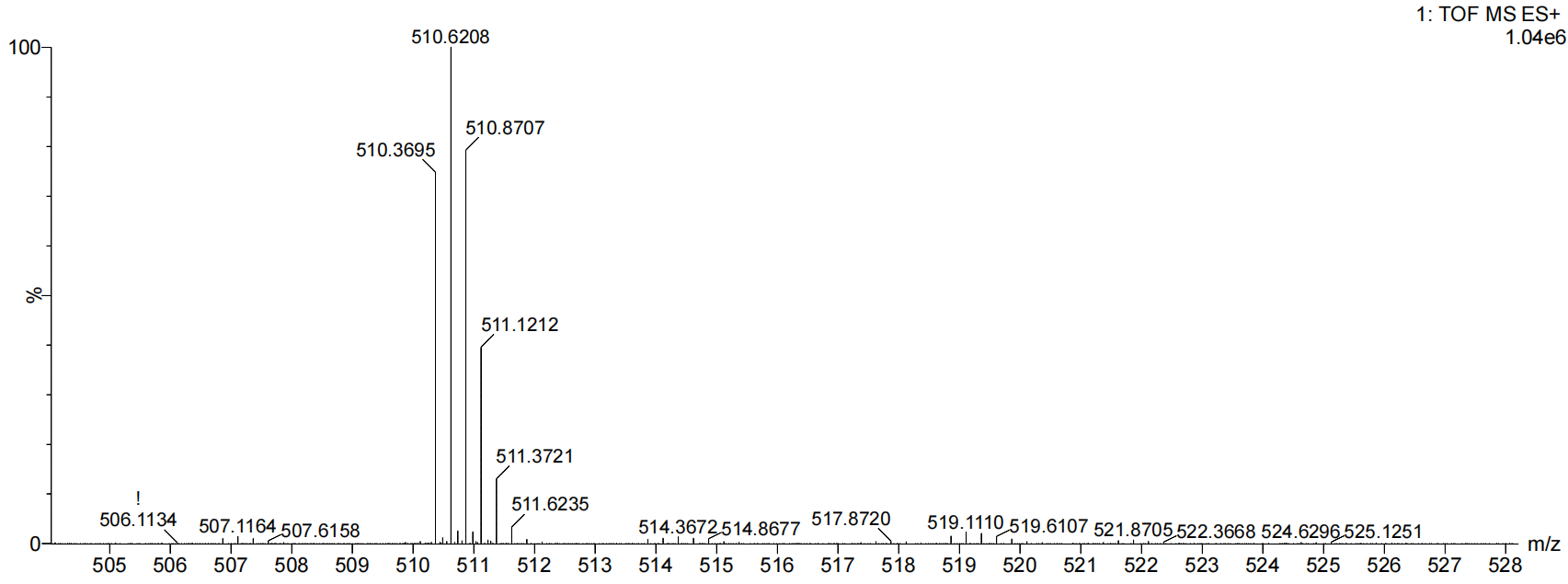


**Figure S2**. High-resolution mass spectra of QAAC4A-12C (ESI-QTOF). Mass-to-charge ratio (m/z) for QAAC4A-12C ([M]^4+^, C_124_H_192_N_12_O_12_^4+^), calculated: 510.3690, found: 510.3695.”

# **3. Supporting results and experimental data**

## **3.1 Characterization of SNT**

**Figure S3**. Zeta potential analysis of SNT and SNT reduced by Na_2_S_2_O_4_ in HEPES buffer (10 mM, pH 7.4). Data are represented as mean ± standard deviation (s.d.) from three independent experiments (*n* = 3).

## **3.2 Binding affinities of SNT with guests**


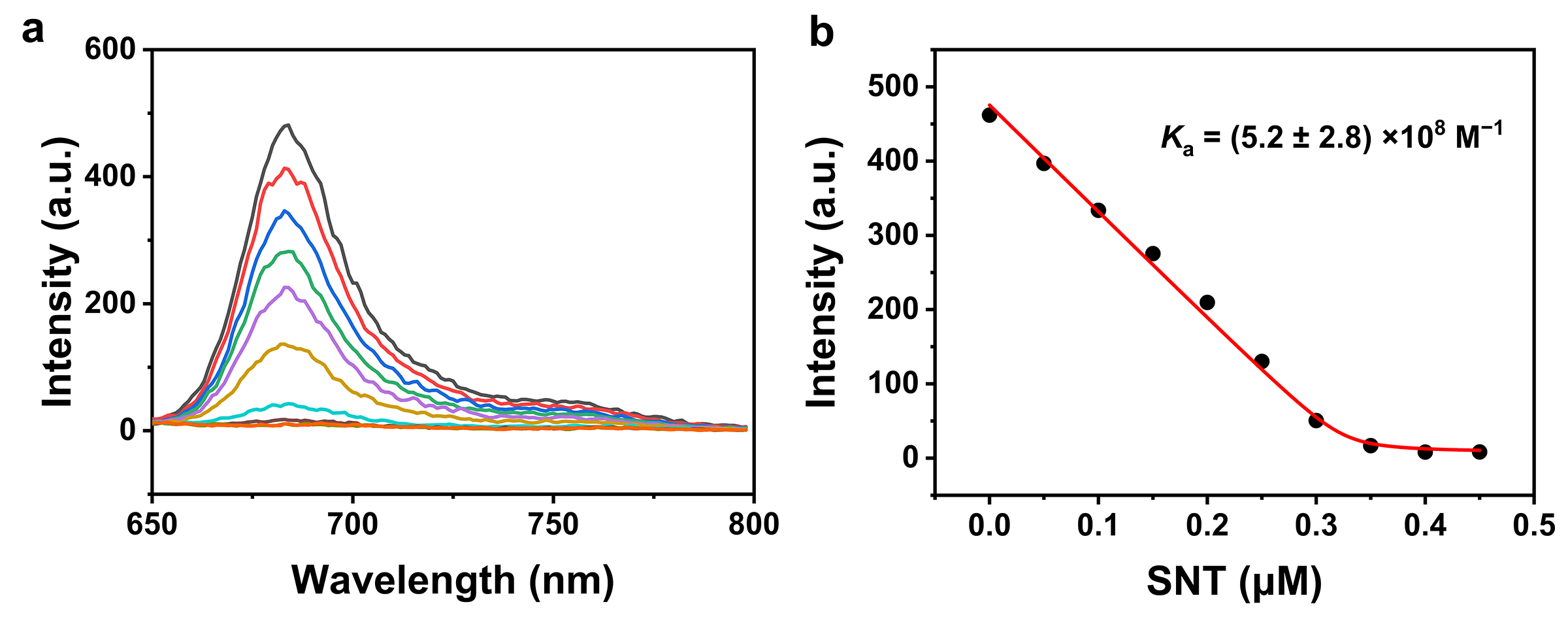


**Figure S4**. **a**) direct fluorescence titration of ZnPcS_4_ (0.6 μM) with SNT (up to 0.45 μM) in HEPES buffer (10 mM, pH 7.4) at 25 ˚C, *λ*_ex_ = 610 nm. **b**) the associated titration curve at *λ*_em_ = 690 nm was fitted according to a 1:2 binding stoichiometry.

## **3.3 Hypoxia responsiveness of SNT–ZnPcS_4_**

**Figure S5**. Fluorescence spectra of ZnPcS_4_ (2.5 μM), NH_2_C4A-12C–ZnPcS_4_ (1.25 μM/2.5 μM), and QAAC4A-12C–ZnPcS_4_ (1.25 μM/2.5 μM) in HEPES buffer (10 mM, pH 7.4) at 25 ˚C, *λ*_ex_ = 610 nm.


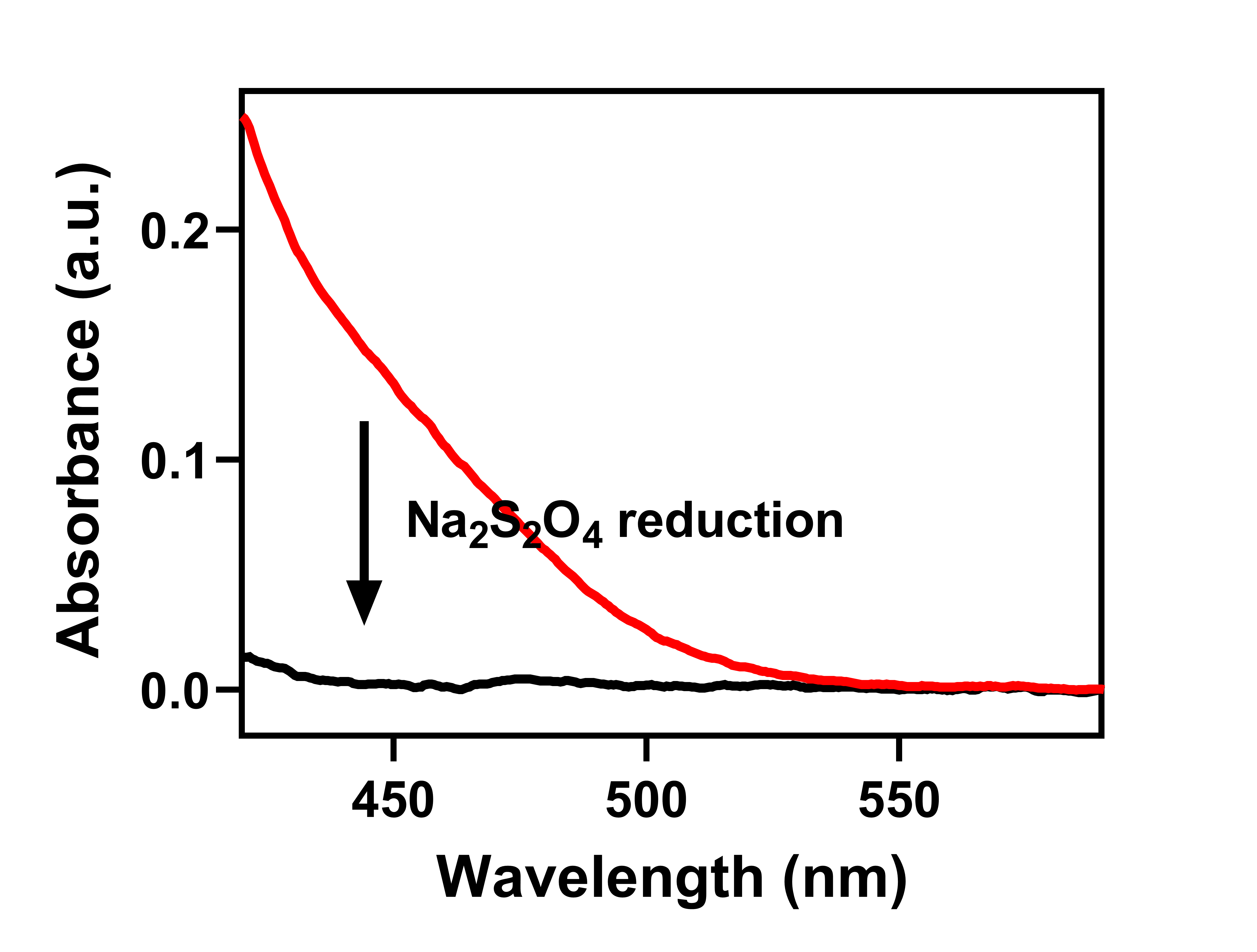


**Figure S6**. Representative UV–vis spectra of SNT before (red line) and after (black line) incubated with Na_2_S_2_O_4_.


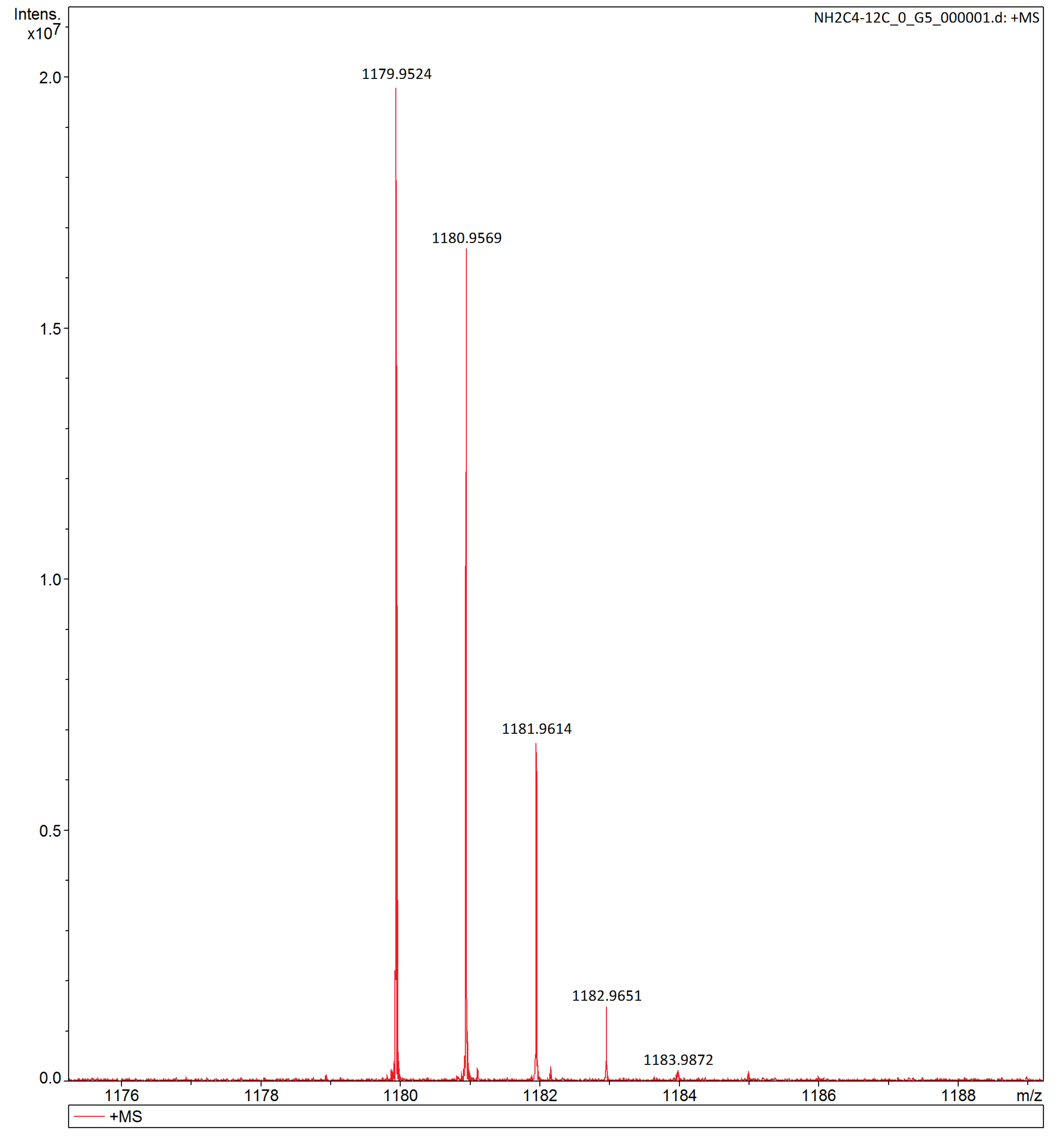


**Figure S7**. Mass spectra of the reduction product after incubating QAAC4A-12C with Na_2_S_2_O_4_ under hypoxic condition at 25 ˚C in HEPES. HRMS (ESI, m/z): calculated for C_76_H_124_N_4_NaO_4_ ([M + Na] ^+^), 1179.9520; found 1179.9524.

**
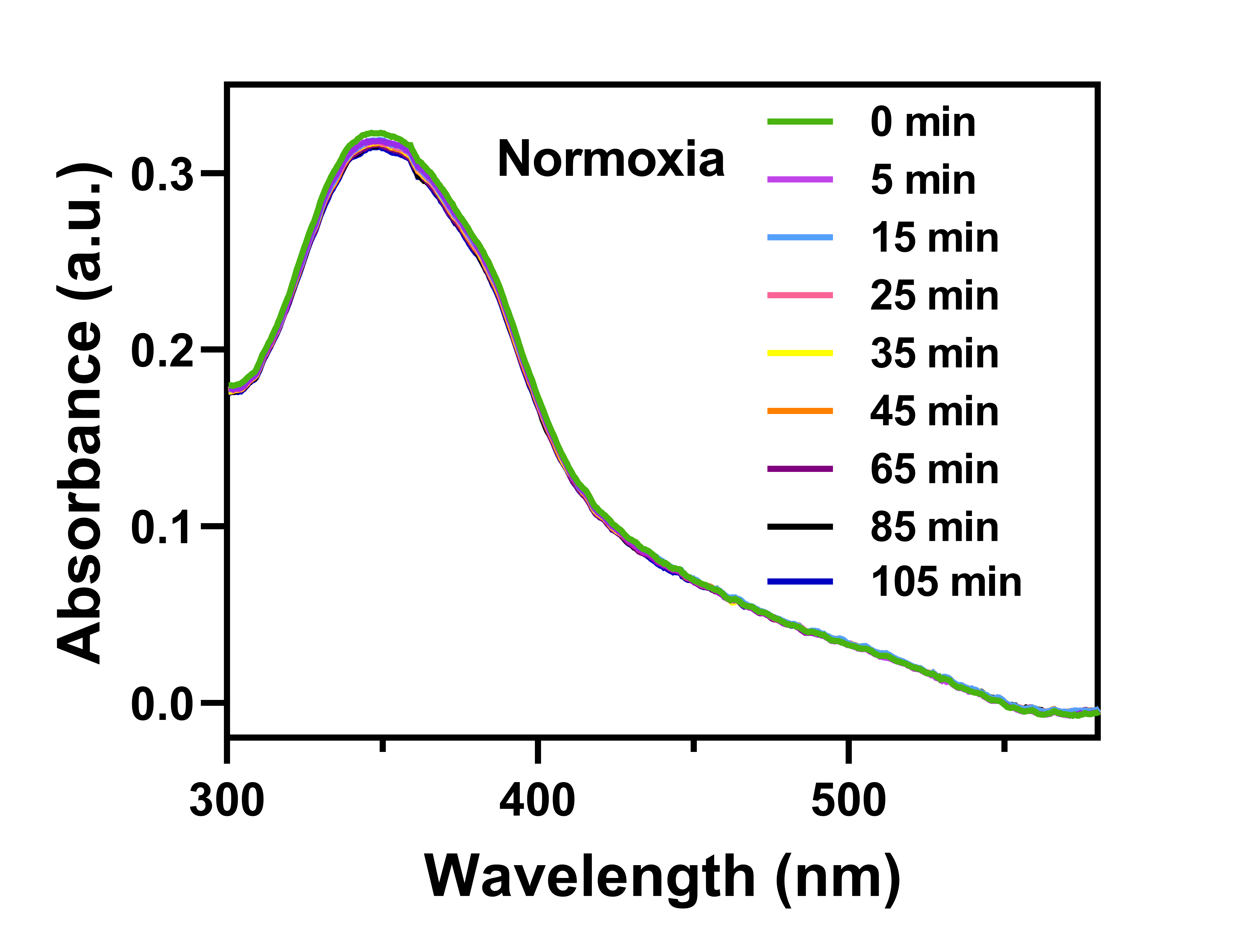
**

**Figure S8**. Absorbance spectra of SNT (4.0 μM) as a function of time following addition with NADPH (10 μM) and DT-diaphorase (0.2 μM) in HEPES (10 mM, pH 7.4) at 37 ˚C under normoxic conditions.


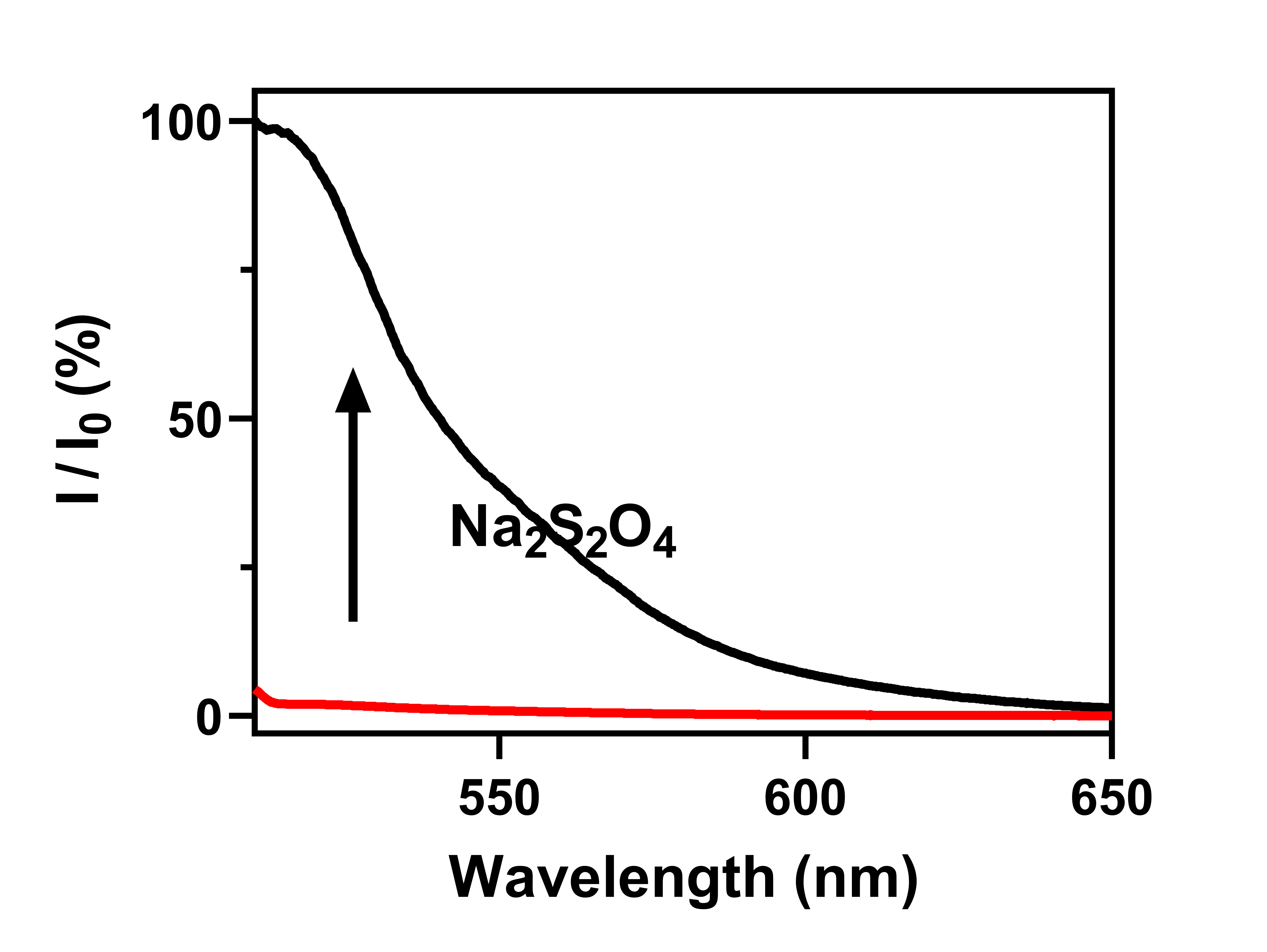


**Figure S9**. The fluorescence spectra of SNT–Fl (4/2 µM) before and after reducing by Na_2_S_2_O_4_ (10 µM).

## **3.4 Synchronous release of the cargo through SNT**


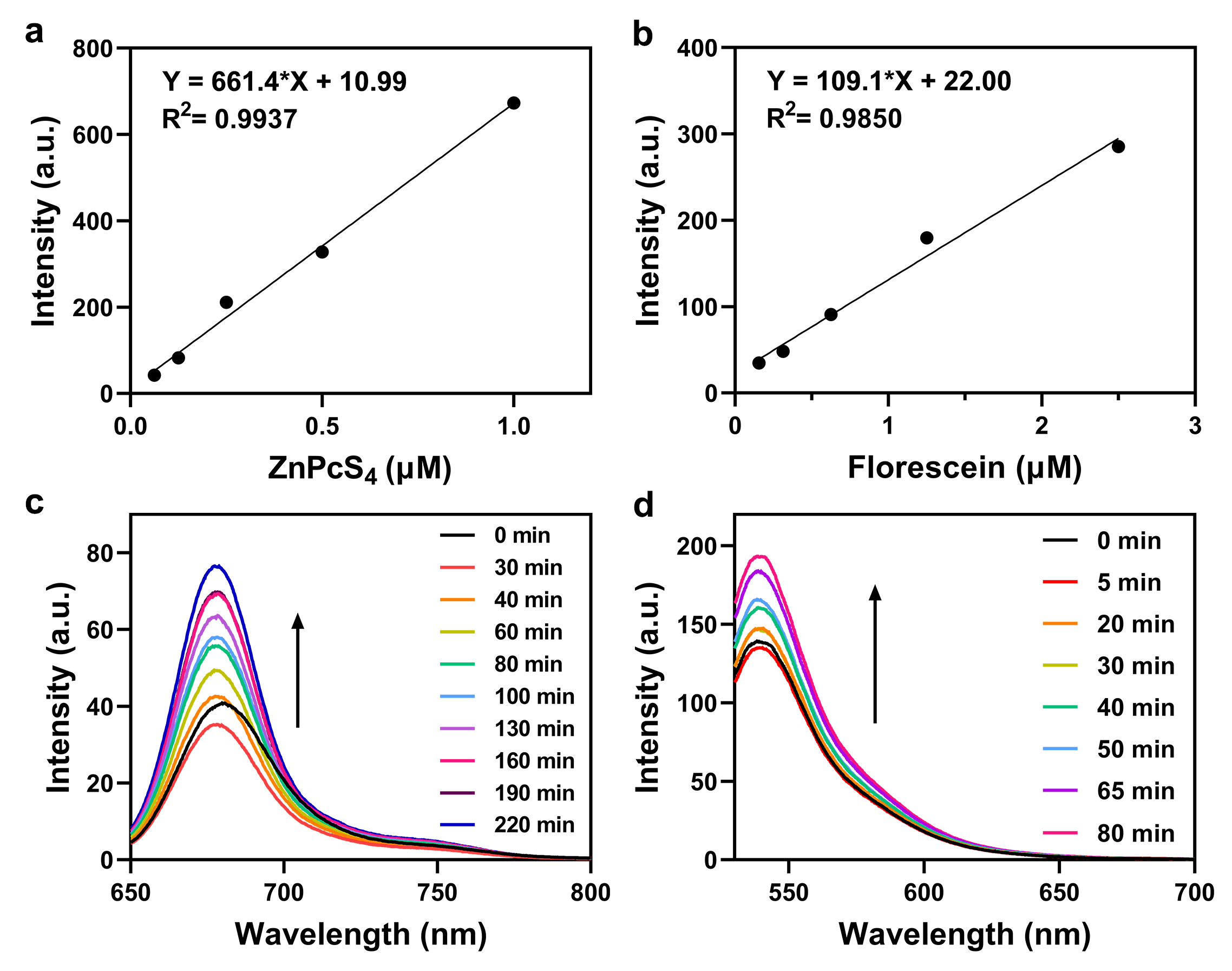


**Figure S10**. **a, b**) The standard curve of ZnPcS_4_ (**a**) and EY (**b**). **c, d**) The release profiles of ZnPcS_4_ (**c**) and EY (**d**) from SNT–Zn/EY as a function of time following addition of DT-diaphorase (0.2 μM) and NADPH (10 μM) in HEPES (10 mM, pH 7.4) at 25 ˚C under hypoxic.

**
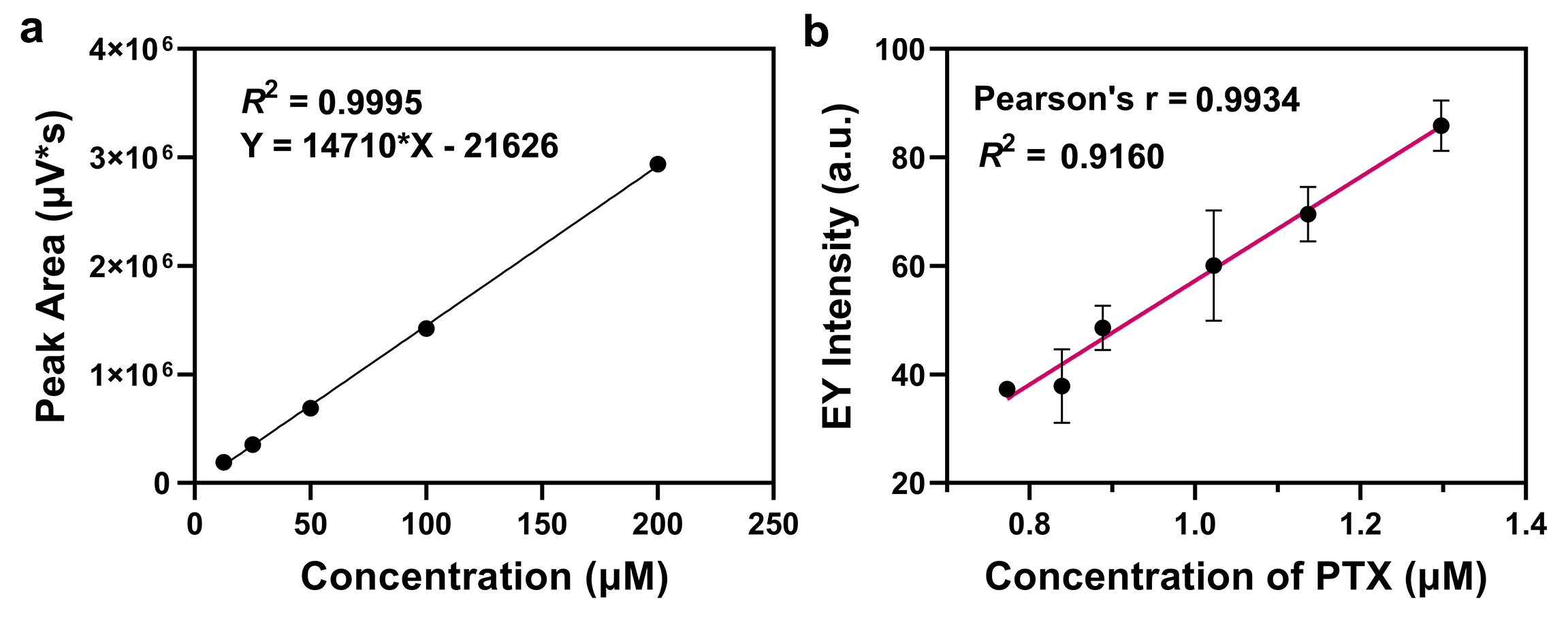
**

**Figure S11**. **a**) The standard curve of PTX by HPLC. **b**) The relationships between the concentration of released PTX and the fluorescence recovery of EY from SNT–EY/PTX (10–5/5 µM) in the presence of NADPH (10 μM) and DT-diaphorase (0.2 μM) in HEPES (10 mM, pH 7.4) at 25 ˚C under hypoxic conditions. Data (b) are represented as mean ± standard deviation (s.d.) from three independent experiments (*n* = 3).

**
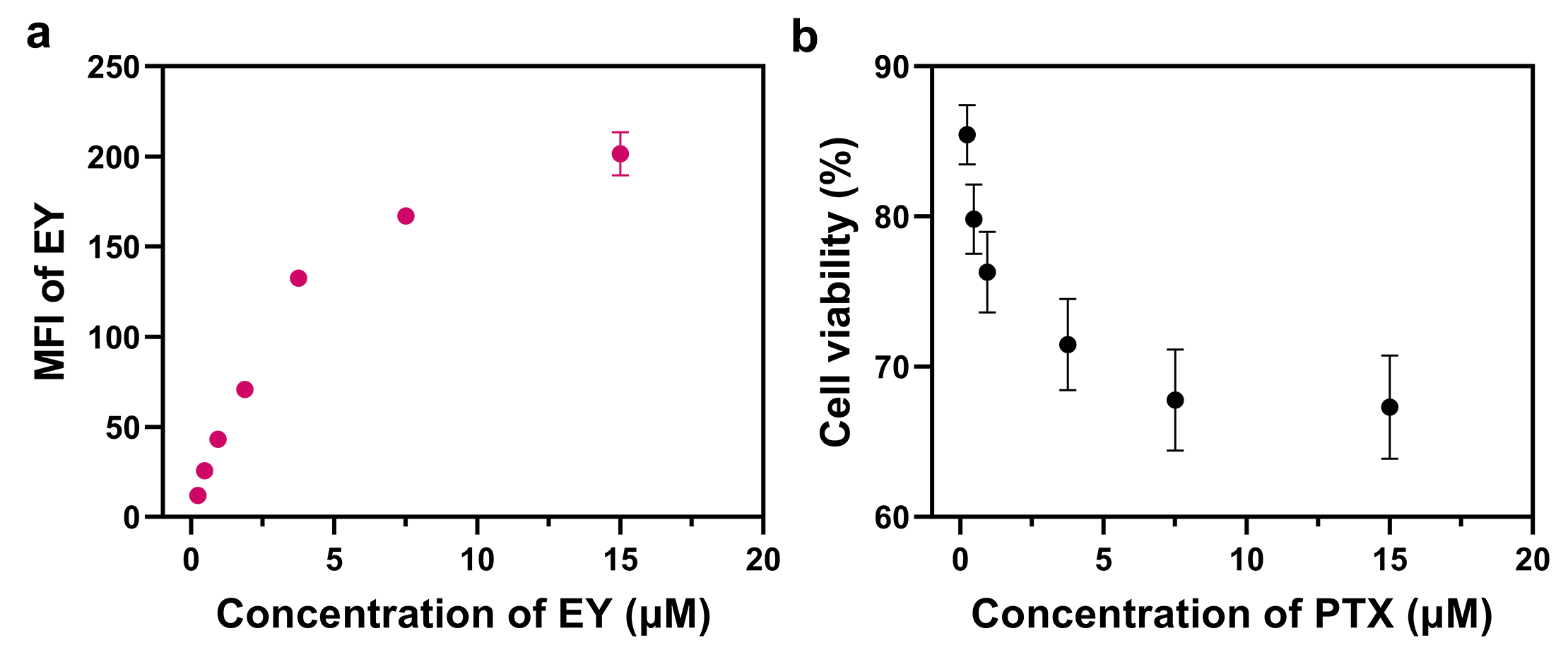
**

**Figure S12**. The mean fluorescence intensity (MFI) (**a**) and the cell viability (**b**) of 4T1 cells after incubation with SNT–EY/PTX under hypoxic conditions for 24 h. Data are represented as mean ± standard deviation (s.d.) from three (**a**) (*n* = 3) and six (**b**) (*n* = 6) independent experiments.

## **3.5 The biodistribution of SNT@F8BT**

**
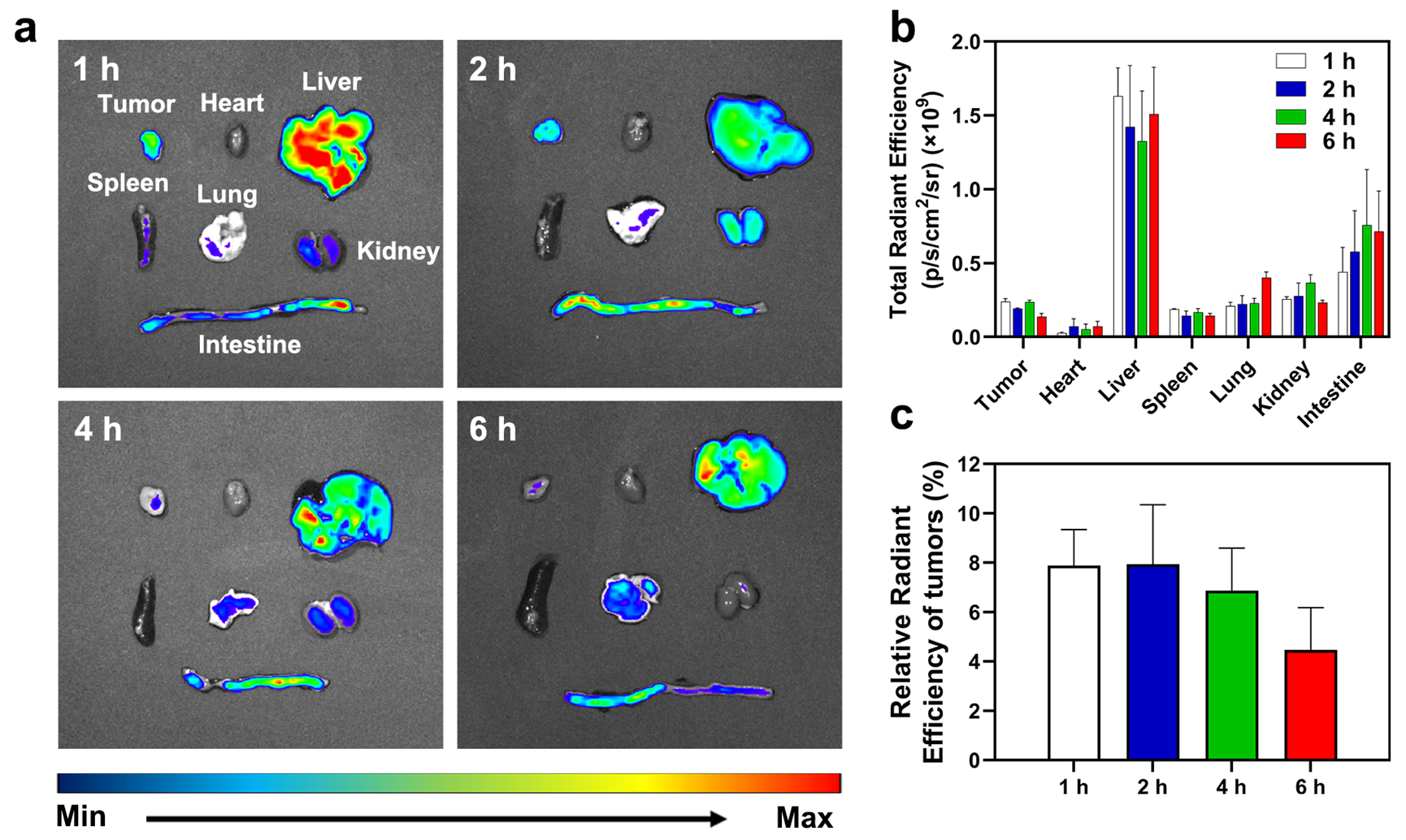
**

**Figure S13**. The biodistribution of SNT@F8BT in 4T1 tumor-bearing mice. Data are represented as mean ± standard deviation (s.d.) from three independent experiments (*n* = 3).

## **3.6 Individual tumor growth kinetics in different groups**


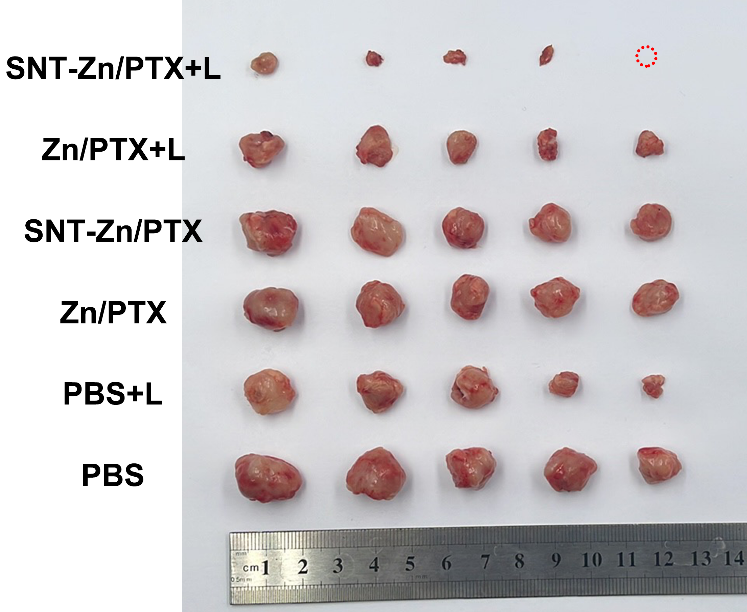


**Figure S14**. Direct observations of tumor tissues harvested from mice treated with PBS, PBS+L, Zn/PTX, Zn/PTX+L, SNT–Zn/PTX, and SNT–Zn/PTX+L (*n* = 5).

## **3.7 Biosafety analysis of SNT–Zn/PTX**

**
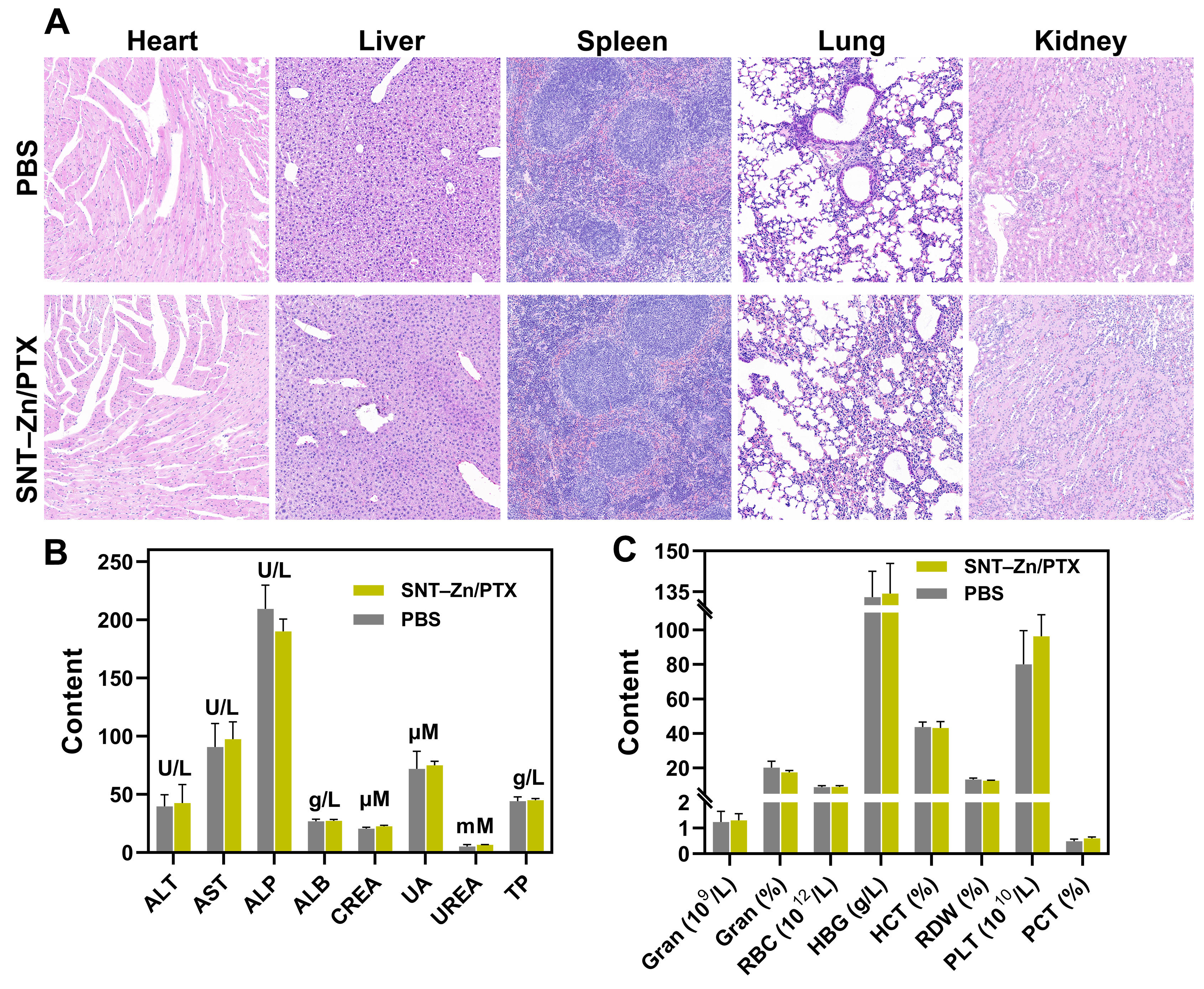
**

**Figure S15**. **a**) Representative H&E images of the main organs from 4T1-bearing mice treated with PBS and SNT–Zn/PTX. The organs were dissected from mice on 14 days after treatment. Blood biochemical analysis (**b**) and blood routine (**c**) from mice treated with PBS or SNT–Zn/PTX on 14 days. Data represents means ± s.d. from three independent replicates (*n* = 3).

## **3.8 Individual tumor growth kinetics in different groups**


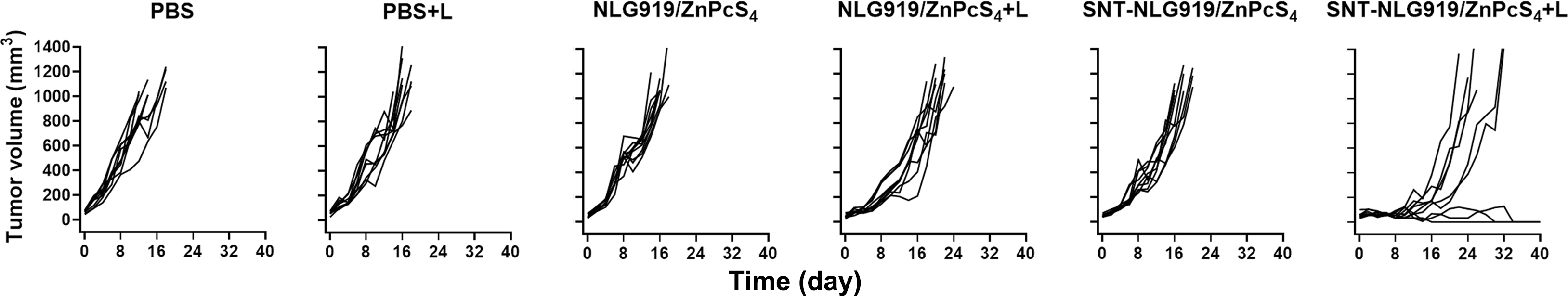


**Figure S16**. Individual tumor growth kinetics from mice treated with PBS, PBS+L, Zn/NLG919, Zn/NLG919+L, SNT–Zn/NLG919 and SNT–Zn/NLG919+L. Growth curves were stopped when the first mouse of the corresponding group died (*n* = 9).

## **3.9 Flow cytometry analysis of cells**


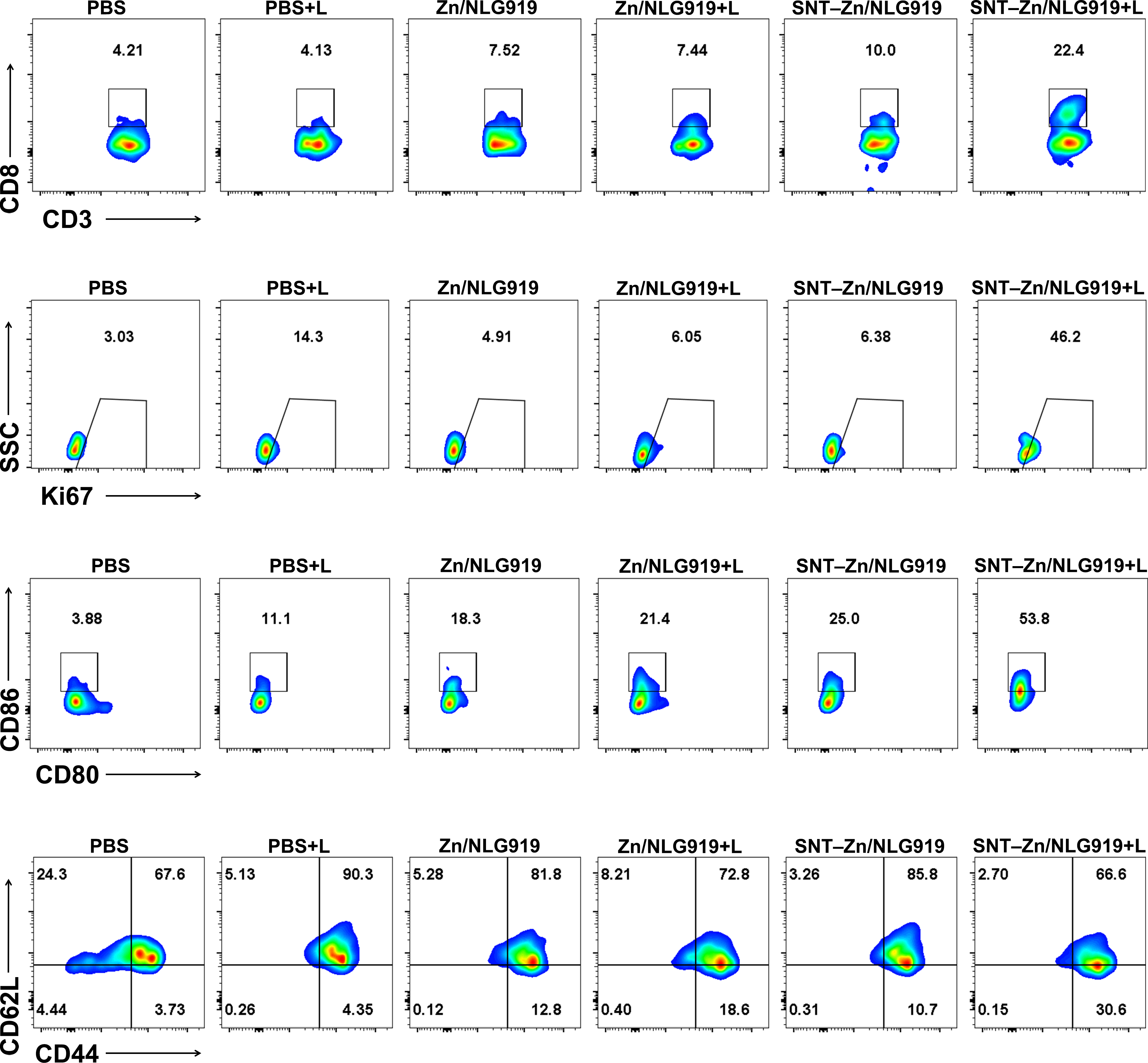


**Figure S17.** Representative flow cytometry plots of Ki67^+^ cells (CD45^+^CD3^+^) within the tumors from the mice treated with PBS, PBS+L, Zn/NLG919, Zn/NLG919+L, SNT–Zn/NLG919 and SNT–Zn/NLG919+L.


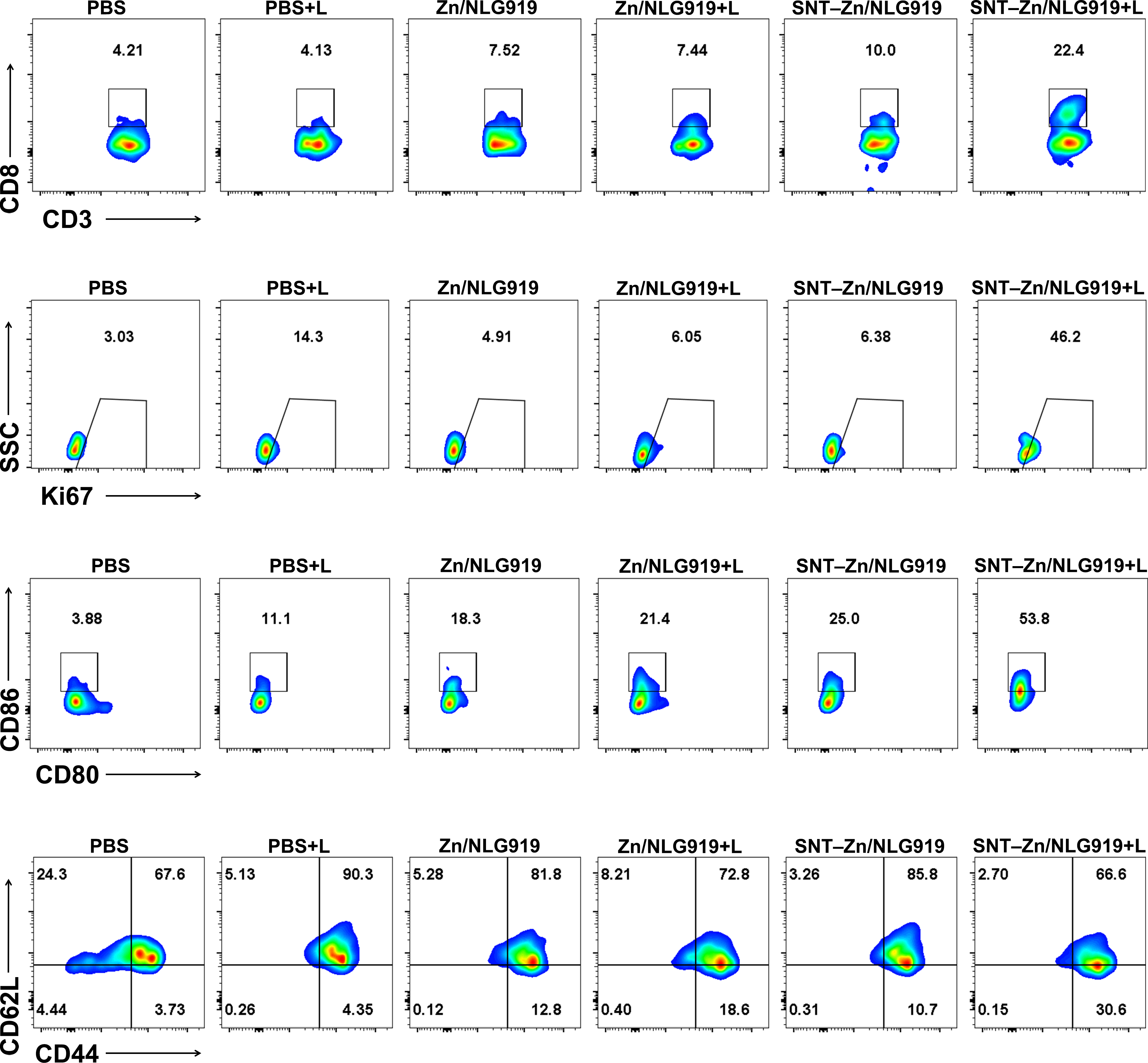


**Figure S18**. Representative flow cytometry plots of splenic memory T cells (CD8^+^CD44^high^CD62L^low^) within the tumors from the mice treated with PBS, PBS+L, Zn/NLG919, Zn/NLG919+L, SNT–Zn/NLG919 and SNT–Zn/NLG919+L.


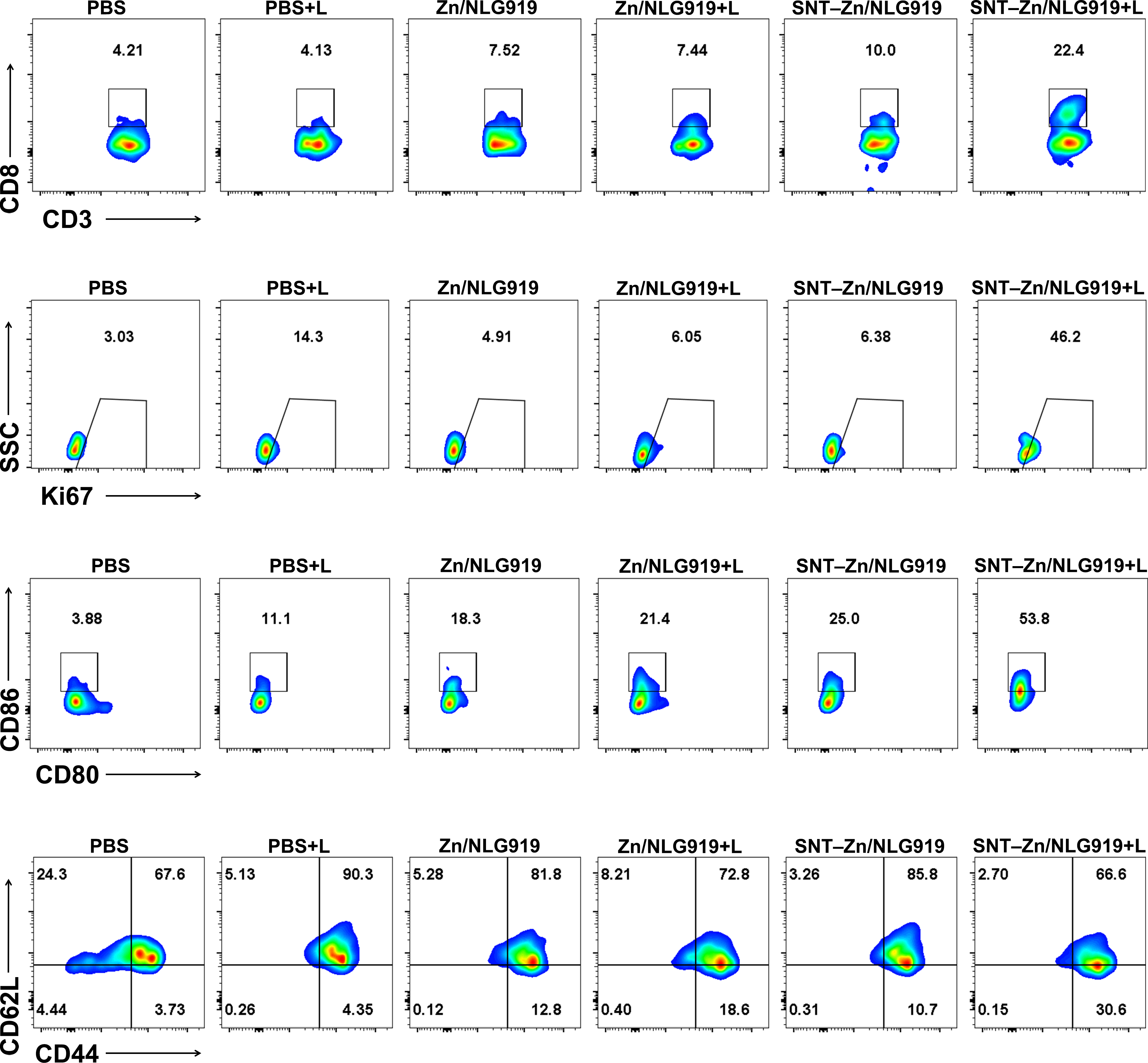


**Figure S19**. Representative flow cytometry plots of DC cells (CD11c^+^CD86^+^CD80^+^) within the tumors from the mice treated with PBS, PBS+L, Zn/NLG919, Zn/NLG919+L, SNT–Zn/NLG919 and SNT–Zn/NLG919+L.

## **Table S1**. Binding affinities of QAAC4A-12C and NH_2_C4A-12C with guest molecules.

| Host | Guest | *K*_a_ (M^-1^) | Property | Loading efficiency | |  |
| --- | --- | --- | --- | --- | --- | --- |
| QAAC4A-12C | EY | (2.6 ± 0.6) × 10^7^ | hydrophilic | | 98% | |
| QAAC4A-12C | Fl | (2.05 ± 0.32) × 10^8^ | hydrophilic | | >99% | |
| QAAC4A-12C | ZnPcS_4_ | (5.2 ± 2.8) × 10^8^ | hydrophilic | | >99% | |
| QAAC4A-12C | MTX | (2.38 ± 0.58) × 10^7^ | hydrophilic | | 98% | |
| QAAC4A-12C | CHL | (6.21 ± 0.26) × 10^7^ | hydrophobic | | 99% | |
| QAAC4A-12C | PTX | (4.81 ± 0.41) × 10^6^ | hydrophobic | | 95% | |
| QAAC4A-12C | NLG919 | (2.23 ± 0.67) × 10^7^ | hydrophobic | | 98% | |
| NH_2_C4A-12C | ZnPcS_4_ | <1000 | hydrophilic | | <8% | |
| NH_2_C4A-12C | EY | <1000 | hydrophilic | | <8% | |

## **Reference:**

[1] W. C. Geng, Y. C. Liu, Y. Y. Wang, Z. Xu, Z. Zheng, C. B. Yang, D. S. Guo, *Chem. Commun. (Camb.)* **2016**, *53*, 392.

[2] D.-S. Guo, V. D. Uzunova, X. Su, Y. Liu, W. M. Nau, *Chem. Sci.* **2011**, *2*, 1722.

[3] a) A. Hennig, H. Bakirci, W. M. Nau, *Nat. Meth.* **2007**, *4*, 629; b) H. Bakirci, W. M. Nau, *Adv. Funct. Mater.* **2006**, *16*, 237.

[4] J. Gao, J. Li, W.-C. Geng, F.-Y. Chen, X. Duan, Z. Zheng, D. Ding, D.-S. Guo, *J. Am. Chem. Soc.* **2018**, *140*, 4945.

[5] P. Shahgaldian, M. A. Sciotti, U. Pieles, *Langmuir* **2008**, *24*, 8522.
